# Convergent evolution of diverse *Bacillus anthracis* outbreak strains toward altered surface oligosaccharides that modulate anthrax pathogenesis

**DOI:** 10.1371/journal.pbio.3001052

**Published:** 2020-12-28

**Authors:** Michael H. Norris, Alexander Kirpich, Andrew P. Bluhm, Diansy Zincke, Ted Hadfield, Jose Miguel Ponciano, Jason K. Blackburn

**Affiliations:** 1 Spatial Epidemiology & Ecology Research Laboratory, Department of Geography, University of Florida, Gainesville, Florida, United States of America; 2 Emerging Pathogens Institute, University of Florida, Gainesville, Florida, United States of America; 3 Department of Population Health Services, Georgia State University, Atlanta, Georgia, United States of America; 4 Department of Biology, University of Florida, Gainesville, Florida, United States of America; HHMI, Massachusetts Institute of Technology, UNITED STATES

## Abstract

*Bacillus anthracis*, a spore-forming gram-positive bacterium, causes anthrax. The external surface of the exosporium is coated with glycosylated proteins. The sugar additions are capped with the unique monosaccharide anthrose. The West African Group (WAG) *B*. *anthracis* have mutations rendering them anthrose deficient. Through genome sequencing, we identified 2 different large chromosomal deletions within the anthrose biosynthetic operon of *B*. *anthracis* strains from Chile and Poland. *In silico* analysis identified an anthrose-deficient strain in the anthrax outbreak among European heroin users. Anthrose-deficient strains are no longer restricted to West Africa so the role of anthrose in physiology and pathogenesis was investigated in *B*. *anthracis* Sterne. Loss of anthrose delayed spore germination and enhanced sporulation. Spores without anthrose were phagocytized at higher rates than spores with anthrose, indicating that anthrose may serve an antiphagocytic function on the spore surface. The anthrose mutant had half the LD_50_ and decreased time to death (TTD) of wild type and complement *B*. *anthracis* Sterne in the A/J mouse model. Following infection, anthrose mutant bacteria were more abundant in the spleen, indicating enhanced dissemination of Sterne anthrose mutant. At low sample sizes in the A/J mouse model, the mortality of Δ*antC*-infected mice challenged by intranasal or subcutaneous routes was 20% greater than wild type. Competitive index (CI) studies indicated that spores without anthrose disseminated to organs more extensively than a complemented mutant. Death process modeling using mouse mortality dynamics suggested that larger sample sizes would lead to significantly higher deaths in anthrose-negative infected animals. The model was tested by infecting *Galleria mellonella* with spores and confirmed the anthrose mutant was significantly more lethal. Vaccination studies in the A/J mouse model showed that the human vaccine protected against high-dose challenges of the nonencapsulated Sterne-based anthrose mutant. This work begins to identify the physiologic and pathogenic consequences of convergent anthrose mutations in *B*. *anthracis*.

## Abstract 2

A study of the spontaneous loss of the spore coat monosaccharide anthrose suggests that convergent evolution in several anthrax strains towards increased pathogenicity could exacerbate global human and animal anthrax disease.

## Introduction

Anthrax, a bacterial disease recognized since ancient times, is caused by the gram-positive spore-former *Bacillus anthracis*. It served as the model organism for establishing Koch’s postulates and its status as a biothreat immediately comes to mind. Soil-borne *B*. *anthracis* spores are associated with specific soil chemistry nearly worldwide and predominately affect grazing domestic and wild ruminants, resulting in high zoonotic mortality and frequent epizootic outbreaks. Spillover to humans is common in many countries, particularly resource-limited countries. We recently modeled the geographic distribution of anthrax globally, identifying 63.5 million poor, rural livestock handlers potentially at risk for disease [1]. Phylogenetically, *B*. *anthracis* is divided into 4 major clades: A, B, C, and D. Group A has spread to all continents (but Antarctica). Other clades are not as widely dispersed. The highest degree of genetic diversity is found in southern Africa and was hypothesized as the origin of anthrax evolution as it represented the only location in the world where strains from clades A and B naturally coexist [2]. *B*. *anthracis* found in Chad were characterized as subgroup A_β_ in the A lineage based on early multi-locus variable number tandem repeat (MLVA) genotyping using 8 markers [3]; later, Mali was added to this group [4]. Then, using 25-marker MLVA genotyping and strains from Cameroon and Chad, West African strains were redefined as the E clade [5]. Once MLVA-25 typed, additional Chadian and Nigerian strains were placed in the E clade, and the larger group was named the West African Group (WAG) [6].

Spores are reported to have a half-life of approximately 100 years [7], and environmental decontamination is not an easy task as evidenced by the decades-long decontamination effort of the small Scottish island of Gruinard [8–10]. In enzootic areas, the most effective control measures are intensive veterinary vaccination programs [11,12]. The most common veterinary vaccine globally is an attenuated viable spore vaccine made from the acapsular *B*. *anthracis* Sterne strain. Although effective, anthrax outbreaks still occur in enzootic areas unless vaccination rates are high and sustained annually. Outbreaks still occur in the United States of America [13], including areas where vaccination reaches livestock, but not wildlife [14]. Our group has shown that sustained livestock anthrax programs reduce human disease [11], and interruptions or reduction in sustained efforts lead to rapid increases in human disease [12].

In the soil environment, the bacterium exists as a dormant spore, waiting for the right signals to permeate through the exosporium layer and initiate germination. Spores are introduced through cutaneous wounds (most common human infection), ingestion (most common livestock infection), injection (fly bites—documented in humans, wildlife, and livestock) or inhalation. In cutaneous lesions, spores germinate extracellularly in the presence of plasma and begin elaborating toxin [15]. Remaining spores are quickly phagocytized by neutrophils independently of toxin or capsule production [16]. Spores germinate inside neutrophils and most are killed; however, phagocytosis of *B*. *anthracis* is associated with increased neutrophil cell death in a toxin and capsule-independent mechanism [17]. This balance is often in favor of the host as cutaneous anthrax infections can self-limit. If uncontrolled, toxin and vegetative bacteria spread through the lymph and blood, leading to systemic infections and host death. In contrast, anthrax spores deposited into the lung undergo limited germination in the absence of host cell contact, and alveolar macrophages, not neutrophils, are the major cell type that control bacterial proliferation in the lung [18]. The site of germination is unclear, but toxin is secreted and detected in blood soon after intranasal infection [19]. Vegetative bacteria and spores are killed by neutrophils and alveolar macrophages, but survivors can cross the epithelial barrier at low efficiency via epithelial cell–assisted traversal [20,21] or, intriguingly, through dendritic cell–mediated dissemination [22]. Dendritic cells are present in lung tissues, and it has been demonstrated that dendritic cells quickly phagocytize spores at high efficiency and transport them to lung-associated lymph nodes within 1.5 hours of infection [23,24]. Dendritic cells are non-microbicidal and sample the environment for antigen, and it has been shown that while *B*. *anthracis* is unable to grow well inside macrophages, replication occurs inside dendritic cells [25]. Interestingly, edema toxin induces dendritic cell maturation and significantly enhances their migration toward the lymph node–homing chemokine MIP-3β [25]. The evidence is mounting that dendritic cell–mediated spore transport to local lymph nodes, during which they are triggered to germinate and begin vegetative cell growth, is an important mechanism of anthrax pathogenesis [22,26].

**Fig 1:**
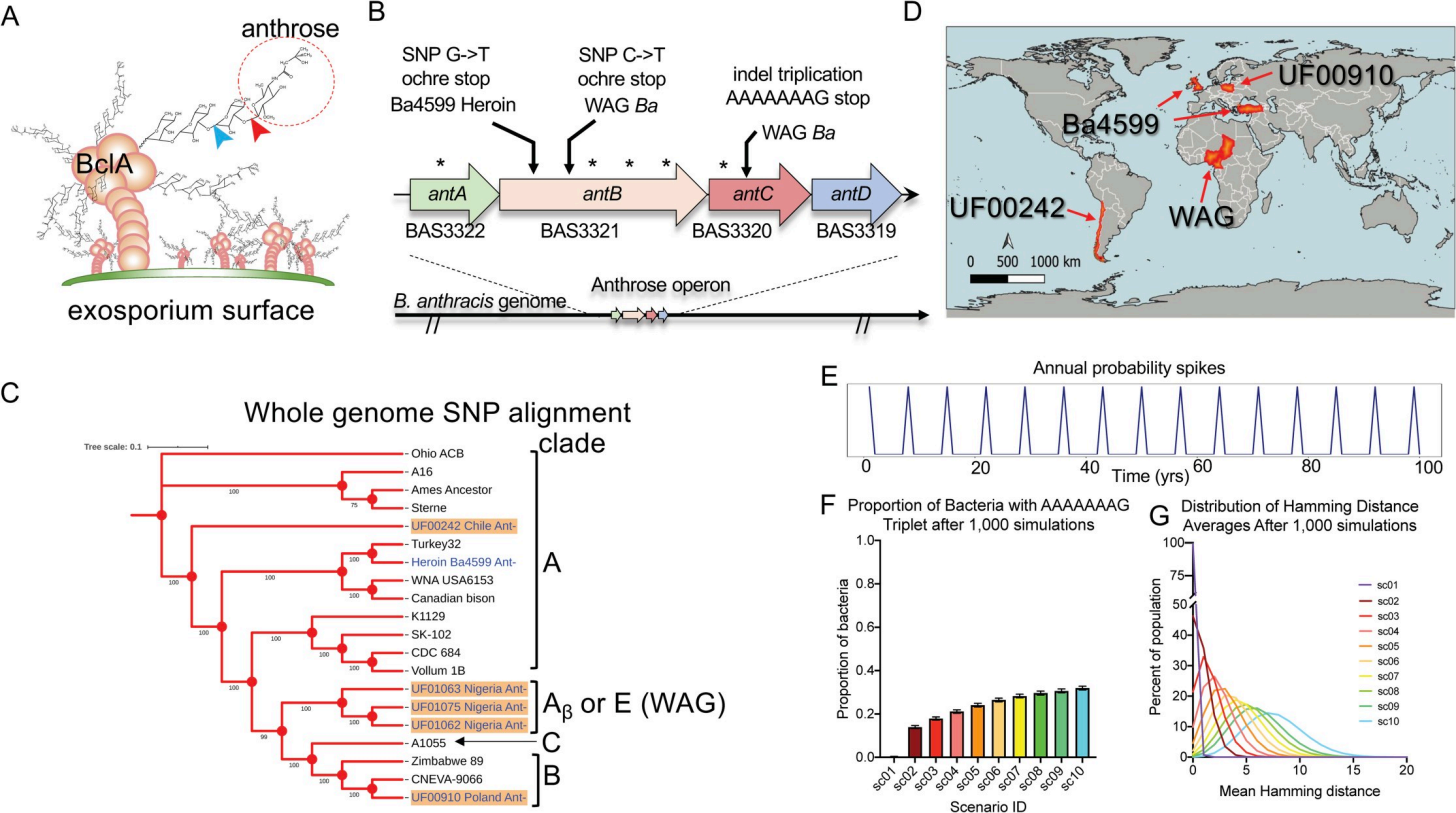
Evidence of convergent evolution toward anthrose-deficient spores. (A) Structure of the anthrose (red circle) monosaccharide shown in relation to the tetrasaccharide glycosylations of BclA and the exosporium surface. The GalNAc residue is not shown, and a number of glycosylations per BclA protein are underrepresented for clarity. Red chevron indicates bonds absent in *antC* or *antD* mutants. Blue chevron indicates bonds not formed in *antB* mutants. Bonds are indicated on 1 tetrasaccharide unit for clarity. (B) The *Ba* Sterne anthrose operon with nonsense mutations indicated by arrows and silent mutations indicated by asterisks found in anthrose-deficient WAG strains and in silico analysis of Ba4599. (C) Whole genome SNP phylogenetic trees representing the phylogenetic relationship of the anthrose-deficient strains to selected type strains in Anthracis lineages. Ant^−^ strains appear in blue type, and strain names with an orange background indicate that they are in the Martin Hugh-Jones Global Anthrax collection. (D) A global map created with QGIS3.8 indicating the countries (in orange) where Ant^−^ *Ba* have originated or caused infections. (E) Mutation probability spikes as a function of time in our in silico simulation of Ant^−^ mutation. (F) Proportion of bacteria presenting with the AAAAAAAG doublet to triplet stop mutation found in WAG strains (gold) after one thousand 600-year simulations at increasing mutation probability scenarios from natural (sc01) to a 1,000 times mutation probability multiplier (sc10). (G) The average number of base pairs different after the triplication event combined with point mutations in the simulated population after the 600-year simulation compared to the wild-type input DNA fragment. The data underlying Fig 1C, 1F and 1G can be found in S1 Data. WAG, West African Group.

The spore surface, or exosporium, is coated with the glycoprotein BclA (Fig 1A). BclA is decorated with a GlcNAc-rhamnose-rhamnose-rhamnose-anthrose pentasaccharide [27,28]. Anthrose is a unique monosaccharide rarely found in nature [29]. The biosynthetic operon is well characterized, and the genes essential for anthrose production have been identified [28,30]. The uniqueness and antigenicity of anthrose identify it as a potential serodiagnostic target [31,32]. BclA is the major component of the exosporium nap layer located on the external face of the exosporium. The BclA-anthrose glycoprotein is involved in spore binding to environmental surfaces, spore hydrophobicity, and spore germination [33–35]. When spores contact a host through ingestion, inhalation, or cutaneous inoculation during the earliest stages of anthrax infection, anthrose is at the host–pathogen interface. The absence of the BclA glycoprotein results in enhanced attachment to epithelial cells [36]. This and other early works did not consider the role of BclA glycosylation status on molecular pathogenesis and did not address pathogen evolution in the wild. A BclA knockout is essentially a triple anthrose, rhamnose, BclA knockout. The importance of glycosylated BclA in targeting spores to integrin CD11b-expressing cells (macrophages and classical dendritic cells) was demonstrated by BclA knockout [37]. Mutational removal of anthrose increased binding of host cell phagocytic receptor CD14 with exosporium rhamnose residues, directly implicating anthrose as an antiphagocytic exosporium residue [38]. Fitness costs of anthrose loss, its role in virulence, and its mechanism in cellular and animal pathogenesis have not been investigated. High levels of immunoglobulin M (IgM) and immunoglobulin G (IgG) specific for anthrose are generated in cattle after Sterne vaccination, demonstrating that anthrose is a major whole-spore vaccine antigen [4]. Registered human vaccines include culture filtrate from toxigenic acapsular vegetative cells (*B*. *anthracis* Sterne strain) where the major protective molecule is protective antigen (PA or PAG). Many studies report that whole-spore vaccines, including the exosporium, are needed to generate full protection to the more virulent *B*. *anthracis* strains [39–42]. Evidence suggests that anthrose is a major Sterne live spore vaccine epitope and is currently being developed as a glycoconjugate vaccine [43–45]. Recently, livestock anthrax cases in heavily vaccinated West Africa were linked to naturally occurring anthrose-deficient strains [6]. The anthrose-deficient strains in West Africa are exclusively in the B. *anthracis* WAG. Until recently, the geography of anthrose-deficient *B*. *anthracis* was restricted to areas in West Africa where outbreaks are frequent, and human mortality rates are among the highest globally [46,47], but sampling for this pathogen is limited [6,48]. Our assay targeting Ant^−^ nonsense mutations identified additional Ant^−^ strains lacking the anthrose biosynthetic genes, one in a divergent clade of the A-group from Chile and another from a B-group strain isolated in Poland (Fig 1B, 1C and 1D; and [49]). Our bioinformatic analysis identified the causative *B*. *anthracis* strain in an anthrax outbreak among European heroin users [50] as anthrose deficient. That outbreak had 28.5% mortality. Whether anthrose deficiency affected mortality rates during the outbreak is uncertain; high numbers of spores were injected by syringe into potentially immunocompromised hosts. The larger picture emerging is representative of several lineages of *B*. *anthracis*, via multiple genetic mechanisms, undergoing convergent evolution toward anthrose deficiency. The slow genetic evolution of *B*. *anthracis* must be kept in mind; the length of time growing in the host is brief compared to the long periods of dormancy outside a host. Whereas Ebola and other bacterial pathogens can evolve before our eyes, *B*. *anthracis* spores lay dormant until host infection. It is estimated that 1.1 billion animals are at risk of anthrax globally, while 198.2 million *B*. *anthracis* Sterne livestock vaccines are administered annually [1]. Loss of anthrose could reduce veterinary vaccine functionality with potential to impact adjacent human populations. Here, we summarize the multiple nonsense mutations resulting in the anthrose-deficient phenotype. Estimated probabilities of anthrose-deficient *B*. *anthracis* evolution in the environment were calculated utilizing computer simulation studies. Using a newly introduced quantitative PCR (qPCR) anthrose SNP typing assay [49], we were able to identify 2 additional anthrose-deficient genotypes in our strain collection, expanding the global locations of anthrose-deficient *B*. *anthracis*. The genomic modifications present in these strains were revealed by whole genome sequencing and verified using traditional PCR. The impact of anthrose-deficient spores on pathogenesis and virulence was investigated by mutational and complementation analysis of *antC*, an anthrose biosynthetic gene, using the capsule-deficient *B*. *anthracis* Sterne vaccine strain. Spore association with epithelial cells and macrophages, infection studies in *Galleria mellonella*, and LD_50_ and mean time to death (TTD) in the A/J mouse model of subcutaneous anthrax were used to assess pathogen fitness gain of anthrose-deficient spores. The mean TTD can be studied with a probabilistic approach that uses Markov processes to model directly the death events as occurring stochastically but according to a hypothesized rate. This approach has been used extensively in many biological settings, from behavioral studies and the estimation of times until predation occurs, to the analysis of the half-life of infectious and epidemic processes and times until an endangered species is quasi-extinct [51–53]. The advantages of such an approach are many: First, as opposed to studying infection processes with ordinary differential equations (ODEs), this approach is more realistic because its units are discrete, not continuous, hence the focus can be put on the fate of individuals. Second, these processes have a long history in biology of being used as flexible instruments to translate fundamental biological hypotheses regarding the speed of a process of interest into testable statistical models that can be confronted with data. Indeed, even fundamental concepts as the modern interpretation of the evolutionary consequences of Mendelism have been recast as stochastic Markov processes [54]. In the context of this study, a class of these Markov processes called “pure death processes” allowed us a process-based cross comparison of exact death rates between the *G*. *mellonella* and mouse infection models that were then extrapolated to larger population sizes commonly observed in natural outbreaks. Finally, the efficacy of the Food and Drug Administration (FDA)-approved human Biothrax Vaccine in this model was investigated.

## Results

### Summary of known Ant^−^ strains and mutations found

In addition to the previously identified nonsense mutations in the *antC* biosynthetic operon, *in silico* analysis of all *B*. *anthracis* genomes in GenBank identified a novel nonsense mutation SNP present in the anthrose biosynthetic gene *antB* of strain Ba4599 [50]. This strain was associated with an anthrax outbreak among injection heroin users in Europe [55,56] (Fig 1B). Additionally, we identified 2 strains in our collection, one from Chile and one from Poland, having neither the wild-type nor mutant SNP. They also failed to produce a PCR product when the anthrose biosynthetic genes were targeted for standard PCR fragment analysis, indicating an absence of these genes. The phylogenetic relationships of these strains to the major clades of *B*. *anthracis* are depicted in Fig 1C, and their source countries are depicted in Fig 1D.

### *In silico* simulation of anthrose operon inactivation

Computer simulation studies were performed to assess possibilities of anthrax mutations over time in the anthrose biosynthetic operon. The goals of the simulation studies were (1) to investigate probabilities of mutation within the coding region of the anthrose biosynthetic operon based on simulated mutation rates extrapolated from previously published molecular clock studies and observed wildlife outbreak frequencies; and (2) to investigate probabilities of whether conversion of Ant^+^ anthrax to Ant^−^ anthrax over a prolonged period of time is solely due to natural mutation or if there is a recent yet unknown selective pressure for Ant^−^ *B*. *anthracis*.

Since *B*. *anthracis* is a spore former, it has 2 very different states. As a spore, it does not multiply and remains dormant in the environment, which can last for a long period of time (years or decades). During vegetative growth, it actively replicates inside the host, which is a relatively short period of time. The number of generations per year is highly variable and is associated with the number of host infections. During enzootic years, host infections are sporadic, but during epizootic years, the number of host infections increases [57]. To capture this feature of anthrax disease in our simulation, 2 different mutation probabilities were incorporated into the simulations: one for enzootic years and one for epizootic years. Within the simulation, every seventh year was assumed to be a year of epizootic anthrax outbreak with much higher mutation, insertion, and deletion probabilities due to increased bacterial growth during years of epizootic outbreaks (Fig 1E).

Estimates of the proportion of wild-type *B*. *anthracis* with the 8-bp AAAAAAAG doublet to triplet nonsense mutation in *antC* by the end of the simulation period are shown in Fig 1F, and the estimates are summarized in S1 Table. Data interpretation for scenario 1 would be as follows: The median estimated proportion of Ant^−^ bacteria is 0.0031 with the 95% confidence interval equal to (0.0021; 0.0047). It follows from Fig 1F and S1 Table that when utilizing mutation probability values documented in the literature, only a small proportion of bacteria 0.0031 (95% CI (0.0021; 0.0047)) is expected to become Ant^−^ by simple sequence repeat (SSR) mutations in *antC* over the simulated time of 600 years. Even if mutation probability increased 100- or 1,000-fold, which is unlikely in the natural environment without the addition of a driving evolutionary pressure, then the proportion of Ant^−^ bacteria would only increase to 0.1394 (95% CI (0.1327; 0.1463)) and to 0.3193 (95% CI (0.3102; 0.3279)), respectively (Fig 1F). In conclusion, the naturally acquired mutation observed in WAG *B*. *anthracis* leading to Ant^−^ mutations are unlikely when using the documented probabilities even though the highest probability of SSR mutations was included in scenario 1. If there was a 100- (scenario 2) or 1,000-fold (scenario 10) increase in anthrax mutation probabilities due to some unexplained ecological selective pressures, beyond the already liberal probabilities utilized in the scenario 1 simulation, the naturally observed SSR becomes more likely (sc02 to sc10; Fig 1G).

Fig 1G and S2 Table provide summaries of the estimated mean population Hamming [58] distance across 10,000 population elements at the end of the simulation period. After being submitted to the triplet mutational round, the sequences were submitted to point mutation effects, and the Hamming distance was used to count the number of different characters between the 2 aligned DNA sequences (i.e., number of base pairs different after the 600-year simulation). The sequences at the end of the simulations were compared with the input sequences to compute the Hamming distances of interest. Estimates in Fig 1G and S2 Table refer to averages over the entire population for each of 1,000 runs and corresponding quantiles across 1,000 runs (each run contains 10,000 DNA sequences). For comparison, the original wild-type fragment length input for the simulation study consisted of 2,000 bp encompassing the region of the anthrose operon where natural mutations are observed. At the 50th percentile of scenario 01 (sc01), which utilized the most liberal baseline mutational probability approximations from the literature, only 0.0076 bp over the 2,000 bp input would be different over the 600-year simulation. The data suggest that, for probabilities documented in the literature and used in sc01 of the simulation study, overall mutation in the anthrose biosynthetic operon is not expected to be observed over the considered simulation length. If we add multipliers stepwise through the scenarios, we approach observations of the bp differences observed in Ant^−^ isolates from West Africa. An alternative, but unlikely, explanation is that pressure for mutations in the anthrose operon have been occurring for a much longer period of time than the 600-year simulation that was run. This then begs the question, why are we finding numerous anthrose-deficient strains now, and why at several locations around the world despite the slow evolutionary rate of *B*. *anthracis*?

### Genomic sequencing of *Bacillus anthracis* strains from Chile and Poland and verification of large genomic deletions

**Fig 2:**
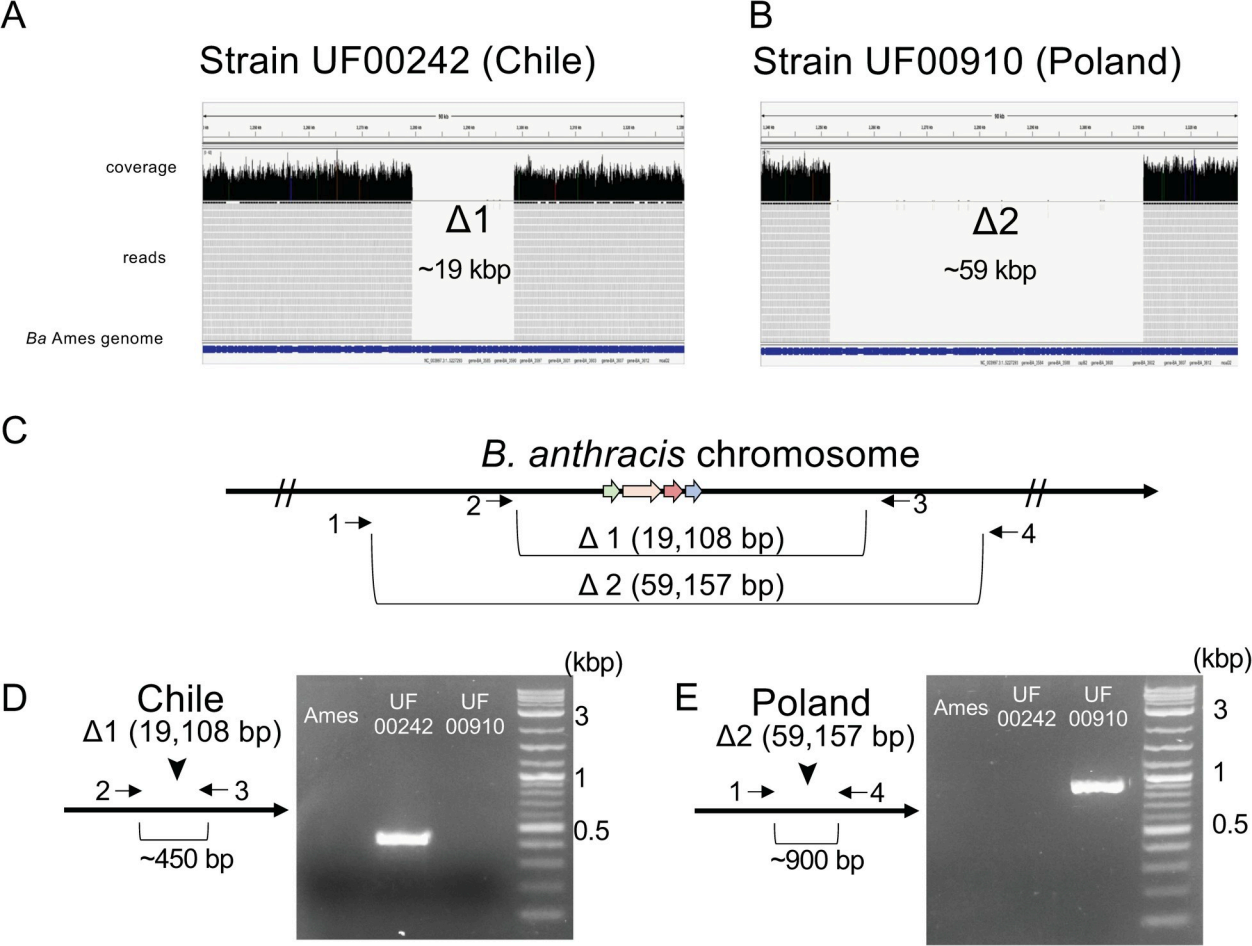
Genome sequencing reveals 2 unique chromosomal deletions encompassing the anthrose operon. (A) Illumina sequence alignment of strain UF00242 and (B) UF00910 to the *Ba* Ames *antABCD* region. The approximate size of the deletions is shown. (C) The exact deletion sizes shown in relation to the *Ba* chromosome with primer sites used to verify deletions indicated by arrows. (D) Agarose gel showing the approximately 450-bp fragment generated from the Chile strain (UF00242) and not in Ames or Polish strain UF00910. (E) Agarose gel showing the approximately 900-bp fragment generated from the Poland strain (UF00910) and not in Ames or Chilean strain UF00242.

Illumina sequencing was performed, and reads were aligned to *B*. *anthracis* Ames. Single large gaps in the read alignment including the *ant* operon and surrounding areas were observed for UF00242 and UF00910 (Fig 2A and 2B). Following de novo assembly with SPAdes, contigs in both contained a spliced region coinciding with the boundaries of the deletion identified by read alignments, confirming the read alignment data. *In silico* MLVA typing of the Chilean *B*. *anthracis* strain verified presence in the A.Br003/004 lineage by MLVA-15, similar to other strains from Argentina. This strain contained a 19,108-bp chromosomal deletion when compared to *B*. *anthracis* Ames. The Polish strain was previously typed as being in group B [59], and in silico MLVA typing verified presence in the B.Br.CNEVA lineage. This strain contained a 59,157-bp deletion centered around the *ant* operon. With strain Ba4599 in the group A1.a lineage, the Chilean strains in the A.Br003/004 lineage, the Polish strain in the B.Br.CNEVA lineage, and the previously published WAG strains in the A_β_/E clade [3,5,6], we have now identified several genetically and geographically distant Ant^−^ strains (Fig 1C and 1D). Primers were designed to amplify small products across the deleted regions present in Chile (UF00242) and Poland (UF00910) Ant^−^ strains (Fig 2C). These primer pairs would give prohibitively large products if the strains had the wild-type genomic material. Primers 2 and 3 were designed to produce an approximately 450-bp fragment present only in the Chilean Ant^−^ isolate. PCR of Ames, UF00242, and UF00910 using primers 2 and 3 resulted in positive PCR product only in the Chilean UF00242 strain, verifying the 19,108-bp deletion found in the de novo sequence and the alignments (Fig 2D). Primers 1 and 4 were designed to produce an approximately 900-bp fragment present only in the Polish strain UF00910. PCR of the strains Ames, UF00242, and UF00910 using primers 1 and 4 resulted in positive PCR product only in the Polish UF00910 strain, further verifying the 59,157-bp deletion found in the de novo sequence assembly and the alignments (Fig 2E).

### Creation of anthrose mutant in *Ba* Sterne and analysis of anthrose content in *Ba* Sterne

Although we have Ant^−^ mutant wild strains, the heterologous strain backgrounds minimize their utility in disentangling the downstream results of anthrose loss, so we removed anthrose from the *B*. *anthracis* Sterne vaccine strain. Sterne lacks pXO2 and so is Cap^−^. The poly-D-glutamic acid capsule is produced by wild-type vegetative bacteria and is required for full virulence but is not involved in sporulation [60,61]. *B*. *anthracis* Sterne sporulates and is Cap^−^Tox^+^, causing a localized infection at the site of inoculation and toxemia-induced mortality in susceptible mice compared to disseminated bacterial growth following infection observed with Cap^+^Tox^-^ *B*. *anthracis* [62,63]. Cap^+^Tox^+^ *B*.*anthracis* generate mortality via toxemia and disseminated bacterial growth. Sterne can cause anthrax disease including lethal factor (LF) and edema factor (EF) toxemia in complement-deficient mouse strains, representing a useful model for understanding the role of anthrose in onset of toxemia-induced mortality [63]. We first created the Δ*antC* mutant by allelic replacement and verified the deletion by PCR (S1A Fig). The absence of anthrose in the spore was verified by gas chromatography–mass spectrometry (GC–MS) retention time comparison (S1B Fig). The unique mass spectrum from the compounds at the observed retention time peaks (S1D Fig) allowed us to propose the structure of the trimethylsilyl (TMS)-derivatized anthrose observed by GC–MS (S1C Fig). The retention time peak doublets at approximately 35 minutes were only present in the pure anthrose and Sterne spore preparations. These peaks corresponded to a unique mass spectrum not in the standard NIST databases. These spectra were not found in the Δ*antC* spore sample verifying functional removal of anthrose in the Δ*antC* spore.

### Physiological and molecular pathogenic characterization of the *Bacillus anthracis* Sterne anthrose deletional mutant

Growth of the strains was carried out in 96 well plates. Sterne 34F2 and the Δ*antC* mutant grew at similar rates and to similar final densities. The complemented mutant exhibited a slight lag most likely due to expression from the plasmid based *antC*. Eventually, all strains reached the same final culture density. Normally, anthrose is produced during late logarithmic growth immediately prior to sporulation, during stage II of the sporulation associated expression wave [28,61]. Artificial expression of *ant* genes from the complementation plasmid throughout lag and log phase growth may dysregulate the natural growth pathways of *B*. *anthracis*.

**Fig 3:**
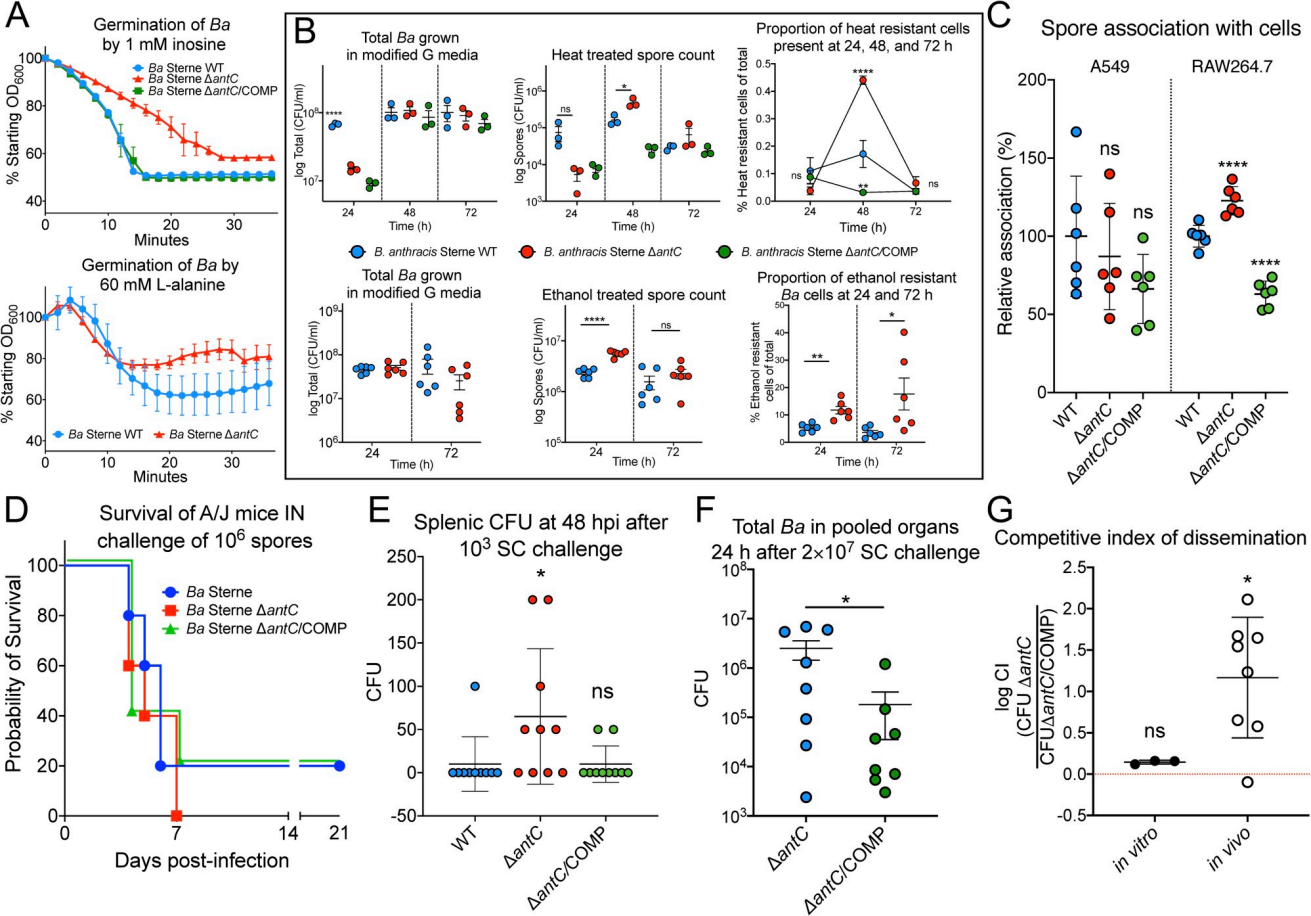
Anthrose deficiency affects *B*. *anthracis* physiology and the pathogenesis of anthrax. (A) *Ba* germination in response to 1 mM inosine as germinant (top) and 60 mM L-alanine (bottom). The experiment was carried out in triplicate for each strain. The OD_600_ read every 2 minutes for 1 hour, and data are presented as percent of starting OD_600_. The symbols represent the mean, and the vertical bars are the standard deviation of the mean at the time points. (B) Sporulation of WT Sterne compared to the Δ*antC* and Δ*antC/*COMP strain as indicated after growth in modified G sporulation media at 24, 48, or 72 hours. The standard error of the mean of 3 heat treatments (top panel) or 6 ethanol treatments per strain are shown (bottom panel). (C) Spore internalization assay using human lung epithelial cell line A549 and mouse macrophage cell line RAW264.7 after infection of spores at an MOI of 10:1. Numbers are internalized spores as a percentage of total spores determined by dilution plating of inoculum and cell lysates. Then the percentages were normalized to WT *Ba* Sterne to obtain relative internalization %. (D) Survival of A/J following intranasal instillation of A/J mice with 10^6^ spores of the indicated strain. (E) In a predetermined endpoint study, mice were infected by the subcutaneous route with 1,000 spores, and spleens were collected 48 hours postinfection. Each dot represents data from a single mouse with mean and standard deviation shown. *p*-Values were calculated by comparing Sterne (blue dots) to Δ*antC* (red dots) and Δ*antC*/COMP (green dots). Splenic bacterial burdens were low, but the levels of Δ*antC* mutant were significantly higher than wild-type and the complemented mutant as calculated by 1-way ANOVA. (F) Total bacterial organ burden of each strain from 8/10 mice challenged with 2 × 10^7^ total spores from the CI study. The means and SEM are shown with significance tested by the Student *t* test. (G) CI values were log transformed, and the standard deviation is shown. The Wilcoxon signed rank test was used to determine the significance of CI difference from 1. *p ≤ 0.05; **p ≤ 0.01; ***p ≤ 0.001; ****p ≤ 0.0001; ns = not significant. The data underlying Fig 3A–3G can be found in S1 Data. CI, competitive index; IN, intranasal challenge; MOI, multiplicity of infection; SC, subcutaneous challenge; WT, wild-type.

Germination is an important physiological step for spore formers. Spore surface molecules, such as anthrose, may affect exosporium permeability to germinants or allow close interaction of germinant signals and exosporium. The germination rates of spores in response to heat and the potent germinant inosine were measured as a decrease in optical density over time. The Sterne vaccine isolate and complement strain had the same germination rates and final spore germination completion levels in response to the germinant inosine (Fig 3A). The Δ*antC* mutant had a slower germination rate and lower percentage of cells successfully germinating in the presence of both inosine and L-alanine, germinants targeting 2 different germination pathways. Sporulation of the Δ*antC* mutant was tested by comparing heat-treating or ethanol treating to untreated aliquots grown in culture. The Δ*antC* mutant produced more heat resistant cells, in terms of total number and relative percentage of total cells, at 48 hours with the wild-type and complement catching up by 72 hours (Fig 3B, top panel). The Δ*antC* mutant produced more ethanol resistant cells at 24 hours (total number and relative percentage of total cells) and 72 hours (relative percentage of total cells) than the wild-type (Fig 3B, bottom panel). Spore association percentages in non-phagocytic A549 human lung epithelial cells were not significantly different (Fig 3C). However, phagocytic RAW264.7 murine macrophages were associated with Sterne Δ*antC* spores at a significantly higher level than wild-type spores and Δ*antC* complement spores (Fig 3C).

### LD_50_ survival, organ CFU, competitive dissemination index, and Biothrax vaccination studies in an anthrax mouse model

**Fig 4:**
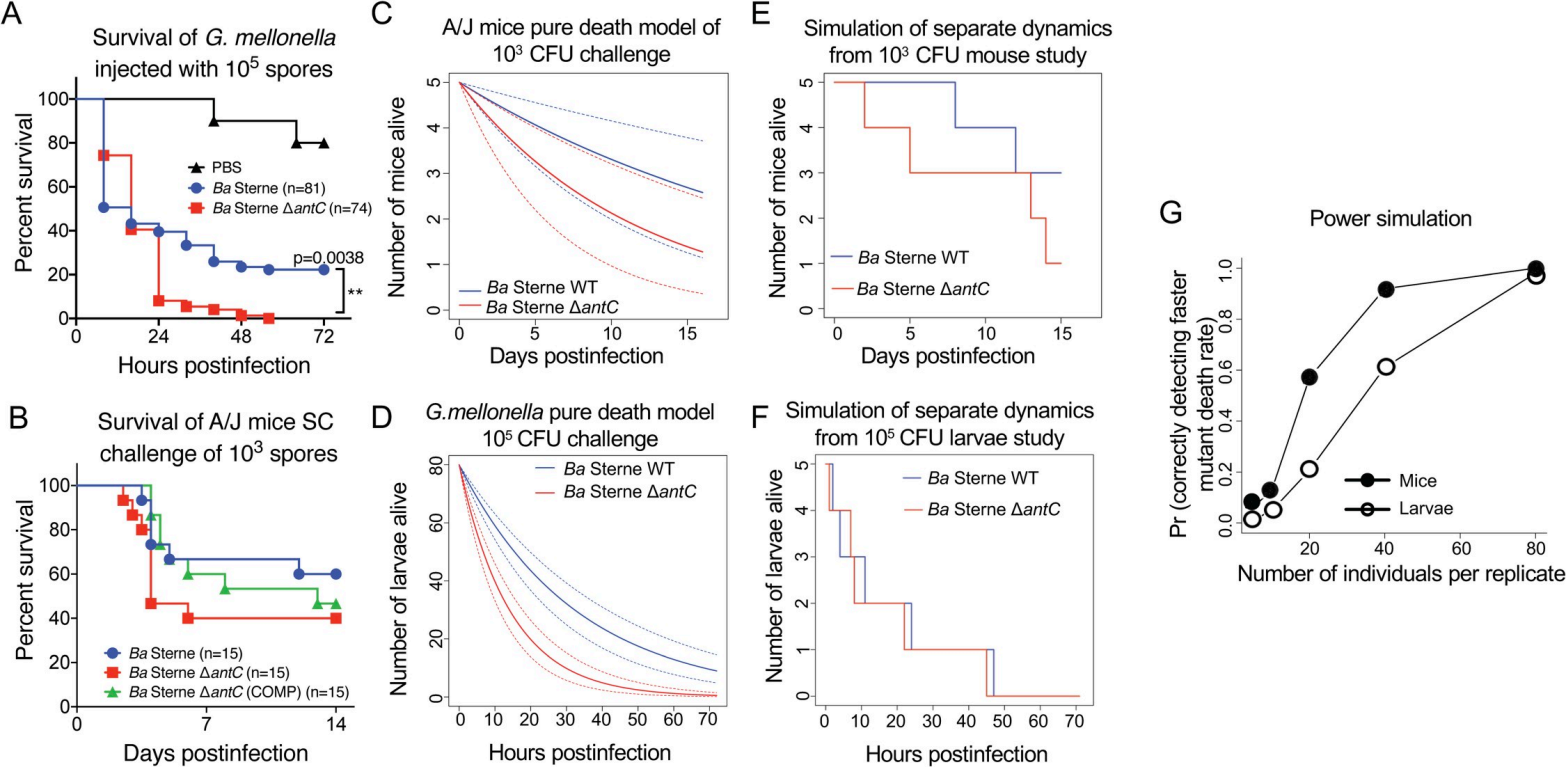
Alternate animal models and pure death process simulations coalesce at increased sample sizes. (A) Subcutaneous infection of A/J mice with 1,000 spores of the indicated strains shows the consistent faster TTD and increased lethality of the Δ*antC* mutant. Data are from 3 independent experiments and 15 challenge mice. (B) Survival of *G*. *mellonella* infected with 10^5^ spores of *Ba* Δ*antC* spores (red squares and line) was significantly different than the *Ba* Sterne wild-type challenged larvae (blue circles and line) as determined by the Mantel–Cox log-rank test. **p < 0.005; *p < 0.05. (C) Plot of the average estimated loss rates in the 1,000 CFU subcutaneous mouse challenge for each strain under the “Separate dynamics model.” (D) Plot of the average estimated loss rates in the 10^5^ CFU *G*. *mellonela* injection challenge for each strain under the “Separate dynamics model.” (E) Simulation of 1,000 CFU dynamics using small sample sizes over the course of a typical animal. (F) Simulation of *G*. *mellonella* death dynamics using small sample sizes. (G) Number of animals per replicate versus the proportion of correctly calling the “Separate dynamics model” using the mouse dynamics (black circles) and the larva dynamics (white circles). The data underlying Fig 4A–4G can be found in S1 Data. CFU, colony-forming unit; SC, subcutaneous challenge; TTD, time to death.

Purified spore colony-forming units (CFUs) were determined and then used to perform an LD_50_ study, survival study, and splenic bacterial organ loading A/J mice challenged via the intranasal or subcutaneous route (S2 Fig). Preparations of spores were visualized under phase contrast microscopy and showed that there was no obvious clumping of spores (S3A Fig). Our results were similar to previously published LD_50_ of Sterne in this model [60]: 60% survival after challenge with wild-type Sterne was observed, and only 40% after challenge with the Δ*antC* mutant (a 20% difference following subcutaneous or intranasal challenge Figs 3D and 4A). Probit regression analysis was used to calculate 7 and 14-day LD_50_ doses (Table 1). At 7 days, the subcutaneous LD_50_ was calculated as 1,810 spores for the wild-type Sterne and 971 spores for the Δ*antC* anthrose mutant, a 53.5% reduction in spore LD_50_. The Sterne Δ*antC* complement had a 7-day LD_50_ at 3,171 spores. The 14-day LD_50_ remained the same for wild-type and Δ*antC* mutant but decreased to 986 spores for the Δ*antC* complement. Mice were more resistant to intranasal challenge but resulted in similar differences in LD_50_. This indicates an increase in mortality and increase in pathogenicity during the early stages of infection with Ant^−^ Sterne isolate. The harmonic mean TTD of 1,000 Δ*antC* mutant spore-challenged A/J mice was 2.76 (66.24 hours) or 2.6 days (62.4 hours) sooner than either the Sterne wild-type or the Δ*antC* mutant complement, respectively. Following subcutaneous inoculation between the shoulders, symptoms such as severe draining lymph node and facial edema occurred markedly sooner in Δ*antC* challenged mice than in wild-type or complement-challenged mice. Upon dissection of mice euthanized 96 hours after subcutaneous challenge, subcutaneous hemorrhaging was more widespread following Δ*antC* spore challenge. Spleens from groups of 10 mice subcutaneously infected with 1,000 spores of the wild-type Sterne, Δ*antC*, or Δ*antC/*COMP were removed and homogenized, and total bacterial CFUs counts were determined (Fig 3E). Overall, the splenic burden was low, as described elsewhere [63]. However, there were significantly more Δ*antC* mutant bacteria present in the spleens of infected animals compared to wild type or complement (approximately 6 times more bacteria). Only 1/10 mice infected with wild-type Sterne had measurable bacterial burden while 6/10 infected with Δ*antC* Sterne did and 2/10 for the Δ*antC*/COMP. Even though mortality in the A/J mouse model occurs primarily by toxemia caused by a localized infection, we found decreased LD_50_, decreased TTD, and earlier onset of symptoms was linked to increased bacterial dissemination of the Δ*antC* mutant. The faster onset of lymph node swelling and increased subcutaneous hemorrhaging support this notion.

**Table 1:** Probit LD_50_ calculations and harmonic mean TTD.

	Probit 7-day LD_50_ (spores)		Probit 14-day LD_50_ (spores)			SC harmonic mean TTD (days)		
Route	IN	SC	IN	SC	Dose	1.0 × 10^6^	1.0 × 10^5^	1.0 × 10^3^
Sterne WT	3.2 × 10^4^	1.8 × 10^3^	3.2 × 10^4^	1.8 × 10^3^		1.76 (1.47–2.21)	4.17 (2.53–11.93)	8.68 (4.45–185.05)
Sterne Δ*antC*	1.5 × 10^4^	9.7 × 10^2^	1.5 × 10^4^	9.7 × 10^2^		1.67 (1.40–2.05)	3.90 (2.38–10.91)	5.92 (3.2–31.84)
Sterne Δ*antC*/COMP	3.5 × 10^5^	3.2 × 10^3^	3.5 × 10^5^	9.9 × 10^2^		2 (2)	5.73 (3.53–15.17)	8.52 (4.92–31.55)

IN, intranasal challenge; SC, subcutaneous challenge; TTD, time to death; WT, wild-type.

A competitive index (CI) study was performed to ascertain if loss of anthrose led to enhanced dissemination of the Δ*antC* mutant. Twenty-four hours after subcutaneous injection of 2 × 10^7^ spores (a mixture of the Δ*antC* mutant and its kanamycin-resistant Δ*antC*/COMP complement) in 10 mice, the pooled organs were homogenized, and total bacterial organ burden and kanamycin-resistant bacterial organ burden were determined. There were significantly higher levels of Δ*antC* mutant bacteria in the organs compared to Δ*antC*/COMP (Fig 3F). In this CI study, a CI > 1 indicates that higher levels of Δ*antC* mutant are present, while a CI < 1 indicates that more Δ*antC*/COMP are present within the organs of each animal, removing confounding effects host variability may have on the bacteria spores with different genetics. The in vitro mean CI was 1.4 (1.22 to 1.58), while the in vivo mean CI was 35.20 (−0.18 to 70.57) (Fig 3G). The Wilcoxon Signed Rank Test found that the in vitro CI data were not significantly different than 1, while the in vivo CI data, though wider ranging, were considered significantly different than 1. This suggests that the loss of anthrose allows the dissemination of more Δ*antC* mutant bacteria in the same host animal. Two of the 10 mice had no detectable bacteria recovered at a detection limit of 100 CFU. One of 10 mice had a CI less than 1, while the other 6 had CIs ranging from 3.799 to 129.4.

Lastly, we wanted to see if loss of anthrose from the spore would result in a reduction of vaccine efficacy in the Sterne A/J mouse model. Groups of 5 unvaccinated challenged mice and vaccinated challenged mice were observed for survival after 20 LD_50_, 50 LD_50_, and 100 LD_50_ subcutaneous challenges (S3B Fig). At all doses, survival of vaccinated animals was 100% with little to no symptoms observed at any point after challenge. Unvaccinated mice succumbed quickly to infection (S3B Fig). These challenge doses cause vaccine failure in mice when using pXO1^+^/pXO2^+^ *B*. *anthracis* [64], indicating the necessity of using fully virulent *B*. *anthracis* strains in future studies with immunocompetent mice.

### Pure death process fitting and simulations

Just like the log-rank tests, the pure death process model fitting and testing only weakly supported the hypothesis that death rates in mice challenged with Ant^**−**^spores were different than in those mice challenged Ant^+^ spores with anthrose due to overlapping confidence intervals (Fig 4A). However, as opposed to the log-rank test, which is designed to detect differences among trends and is based on a chi-squared approximation to the distribution of the test statistic [65], the death process modeling framework was easily amenable to showing that the lack of strength of the results was a small sample size issue, rather than an intrinsic property of the mortalities due to both the wild type and the mutant strain. Indeed, as Dennis and colleagues recently show in their extensive review of classic error statistics in science in a journal issue dedicated to statistical science, classical error statistics do not really compare multiple models simultaneously [66]. With Fisherian significance tests, a single model is tested, and if rejected, one of many plausible alternatives is inferred. Neyman–Pearson tests like the log-rank test appear to compare 2 models but are really significance tests constructed along the axis most likely to reject the “Null” if the “Alternative” is true. Significance tests are calculated as if the null hypothesis were true. Philosophically, this is awkward because as George Box wrote, “All models are wrong.” If so, one may question the gains of doing a significance test. If the test rejects the Null hypothesis, the researcher has not learned anything since it is known a priori that this model is wrong. If the test fails to reject, it only tells that the researcher does not have sufficient sample size or specified inappropriate effect size. Model comparison via consistent information criteria like the Bayesian Information Criterion (BIC) used here, coupled with process simulations, can and do remedy these problems [66,67].

In particular, after first estimating the mouse mortality rate using death process modeling (Fig 4C) under the hypothesis that the mutant dynamics was indeed faster than the wild-type, we simulated 1,000 realizations of the death process using these parameter estimates starting at varying initial sample sizes (i.e., the number of initially challenged mice). Thus, each 1,000 group of simulations contained 5, 10, 20, 40, or 80 challenged mice. One such simulation of small sample sizes is shown in Fig 4E using the mouse dynamics and in Fig 4F using the *G*. *mellonella* dynamics modeled in Fig 4D. For each simulation at each sample size, we tested using the BIC whether the “separate” or the “joint” dynamics model was better and recorded the proportion of times the “Separate dynamics model” was best, given it was the model used to simulate the data. In other words, for each sample size, we estimated the proportion of time the BIC correctly chose the Separate dynamics model as the best model. By doing so, we were able to infer desirable sample sizes needed to detect a higher death rate in the mutant strains when it actually is of the size estimated for the 1,000 CFUs assays. The proportion of time the BIC test correctly chose the Separate dynamics model (akin to a power curve) is plotted in Fig 4G.

Out of each set of simulations, we computed the proportion of cases where a significant difference using BIC was detected when it truly was there. That proportion is an estimate of the detection power of the test. We found that the true difference between survival of Δ*antC* and wild-type challenged mouse groups was correctly detected 6%, 13%, 57%, 92%, and 100% of the time for the initial sample sizes of 5, 10, 20, 40, and 80, respectively. These simulations strongly suggest that larger sample sizes would lead to significantly higher deaths in anthrose-negative *B*. *anthracis* infected animals (Fig 4G). As single epizootic outbreaks in wildlife and livestock are generally larger than the number of animals used in the mouse infection study (*n* = 15) or those in the infection simulation study (3 groups of 20, *n* = 60) [68], outbreaks of anthrose-deficient *B*. *anthracis* in the wild would yield significantly higher mortality rates than anthrose-positive strains when using these animal numbers as a baseline in a predictive epizootic model.

### Infection of *Galleria mellonella* with anthrax spores

The death process analysis approach involved estimation from experimental values followed by power estimation via simulations. Using the estimates from the simulations, the study was repeated by experimentally infecting *G*. *mellonella* larvae with *B*. *anthracis* spores. We hypothesized that using the numbers generated by the power test using the mouse infection variables, anthrose would significantly impact infection in the *G*. *mellonella* model. Using the *G*. *mellonella* infection model, we found that a dose of 10^5^ Δ*antC* spores killed the larvae faster, producing significantly different survival curves (Fig 4B). The Δ*antC* mutant killed 100% of larvae by 56 hours postinfection, while approximately 22% of wild-type Sterne infected larvae survived to the 72-hour study endpoint. The dynamics generated in the *G*. *mellonella* challenge model were utilized in a down-sampling death process simulation to demonstrate the effects low sample size would have had on mortality observations without adequate sample size (Fig 4F).

From a biological standpoint, we consider the *G*. *mellonella* infection model suitable for our inferences because it has been used to characterize major *B*. *anthracis* Sterne virulence factors following vegetative cell challenge [69] and for down-selecting antibacterial peptides active against *Ba* for future use in an animal model [70]. It also allows challenge of desired animal numbers inexpensively and humanely while serving as an accurate innate immune animal model. *G*. *mellonella* possess several components mimicking the mammalian innate immune response. For example, phagocytic hemocytes and production of antimicrobial peptides and reactive oxygen species are triggered within the hemocoel in response to bacterial infections.

## Discussion

The prominent spore saccharide, anthrose, coats the outside of the *B*. *anthracis* exosporium and directs interactions with the environment and host cells. For this work, we focused on the discovery of and consequence of anthrose loss on interactions with host cells. We verified in 2 different strains deletion of large chromosomal regions including the entire anthrose coding region of gDNA in addition to previously published anthrose inactivating nonsense mutations and insertions. Given the multitude of observed mutations resulting in the Ant^**−**^phenotype in diverse strains from around the world, it can be assumed that pressures for removing anthrose are widespread and linked to enhanced survival in the environment or host. Mutations in the anthrose operon were simulated in silico over the course of 600 years, and mutations were found to occur at a low probability, even if outbreak intensities were assumed to occur in many thousands of animals each year. Only when external pressures artificially increased mutational probabilities by 100 to 1,000 times were mutations in the anthrose operon appreciable in our simulation, suggesting an uncharacterized pressure may be contributing to Ant^−^ *B*. *anthracis* emergence.

We characterized the physiological and pathogenic consequences of anthrose-deficient *B*. *anthracis* spores to help us identify these pressures. Removal of anthrose did not impact vegetative growth and enhanced spore formation of *B*. *anthracis*, but germination of the spores in response to the germinants L-alanine and inosine was significantly delayed and incomplete. Modification of the spore surface saccharide content could affect permeability as well as hydrophobicity of the spore surface; neither of which were tested in the present work. Modifying the spore surface could enhance spore clumping; however, this phenomenon was not observed in simple spore suspensions as used for many of our studies. The negative charge of the spore surface is attributed to carboxylic acid groups that are at least partially dependent on the presence of BclA [71]. Anthrose has an unprotonated carboxylic acid group at neutral pH and could be a major contributor to negative spore charge due to the numerous glycosylations of BclA. Removal of that charge and revealing the rhamnose residues below would greatly influence the physical properties of the spore surface and interactions with it, both biological and physical. Furthermore, the association of Ant^−^ spores with murine macrophages was elevated compared to spores with Ant^+^, and no differences were observed when using epithelial cells, indicating that anthrose instills antiphagocytic properties to the spore and is not involved in epithelial cell binding. Anthrose may modulate clumping on the surface of host cells. This would be a result of host–pathogen interaction, and any differences in molecular interactions with the host cell membranes are of interest in future investigations. Previous work [28] noted no differences in growth or sporulation of Sterne anthrose mutants, but the data were not shown, and if made on casual observations of sporulating cultures, differences would be unnoticed. Our experiments used kinetic observations of germination and multiple data points and methods for characterizing sporulation. It is worth noting that, while simpler, heat treatment as a method to enumerate spores likely undercounts the true number of spores in a sample compared to ethanol treatment. These observations reflect other findings, where spores were efficiently recovered from diagnostic samples after ethanol treatment but not heat treatment [72,73]. Observations indicate that spores without anthrose associate more closely with phagocytes and would delay germination if phagocytized. At the site of infection, macrophages could be overwhelmed if more spores are phagocytized, germinate, and secrete toxin. Dendritic cells play an important role in spore germination and bacterial dissemination in anthrax pathogenesis [22]. It is possible that dendritic cells internalize more Ant^−^ spores and migrate farther before toxin secretion could impair host cell integrity due to the germination delay. Cap^−^/Tox^+^ strains like Sterne create a localized infection; however, we found many signatures of modest dissemination enhancement. EF enhances dendritic cell migration toward lymph node–homing chemokine MIP-3β, so higher levels of toxin secretion as a by-product of increased spore phagocytosis, in the near term, could drive dendritic cells further into the lymphoid tissues, decreasing time to toxemia. Approximately 20% more mice died when challenged with Δ*antC* spores regardless of the infection route. The pure death process model dynamics and powers simulation of 60 animals showed that anthrose-deficient *B*. *anthracis* would cause significantly higher population mortality 57% of the time (Fig 4G). Using these numbers as a baseline in the *G*. *mellonella* infection model, the Δ*antC* mutant was significantly more lethal with approximately 20% difference in surviving larvae compared to wild-type *B*. *anthracis* Sterne. The data suggest the decreased lethal dose, earlier TTD, and earlier time to symptoms of infected mice could be caused by enhanced phagocyte uptake coupled with a germination delay. The impact on phagocytes would be biphasic. Macrophages and neutrophils could be overloaded with spores and killed by toxin when they germinate enhancing localized infections. On the other hand, dendritic cells could take up many spores and start migrating to lymph nodes before the spores germinate. Migration to lymphoid tissues would be enhanced by increased ET secretion and increased toxin-mediated cell death in the immune cell–rich tissues. Furthermore, germination delay has been shown to decrease LD_50_s and exacerbate outcomes of mouse infections [74,75]. The cause of this may be the delay of germination initiation, not necessarily the extent of germination, indicating that the delay of germination initiation may allow for more spores to survive the host innate immune response. Enhanced sporulation together with increased virulence may be driving the environmental emergence of anthrose-deficient virulent *B*. *anthracis*. CI of dissemination studies supported the role of anthrose in increased virulence. On average, bacteria descended from anthrose-deficient spores at the time of subcutaneous injection were found at significantly higher levels in the organs of challenged animals 24 hours postinfection. Future testing of germination and sporulation in fully virulent *B*. *anthracis* is currently planned to characterize sporulation and germination in naturally evolving anthrose-deficient *B*. *anthracis*.

A decreased LD_50_, faster TTD, and increased dissemination efficiency would be the basis for a reduction in vaccine efficacy. In the model utilized in this work, a reduction in vaccine efficacy was not observed. We were unable to vaccinate with Sterne (the dominant vaccine strain globally) in this model because the mice are very vulnerable to Sterne. Reasons for this may be 2-fold necessitating further investigation. (1) Complement-deficient A/J mice are susceptible to pXO2^−^ *B*. *anthracis* (i.e., Sterne) because the pXO2-encoded poly-γ-D-glutamic acid capsule, normally required to prevent complement deposition, is not needed. In this model, lethality in anthrax is entirely dependent on toxin production. Toxin levels are dependent on bacterial numbers, and it is likely that the capsule provides an additional in vivo survival advantage beyond prevention of complement deposition. For A/J mice, the subcutaneous LD_50_ of pXO2^+^ *B*. *anthracis* Vollum is 5.5 spores, whereas Sterne is approximately 1,000 spores, supporting a protective role of the capsule beyond prevention of complement deposition on the cell surface such as antiphagocytic properties, pro-inflammatory cytokine induction, and intracellular survival [76,77]. The doses we used (100× LD_50_) should cause a reduction in vaccine efficacy, but none was observed presumably due to the generation of antibodies to PA. (2) The major antigen of the culture filtrate precipitate BioThrax vaccine is secreted PA. The vaccine elicits antibody responses to PA, preventing the binding of PA to host cells, neutralizing its activity, and effectively nullifying all major virulence factors of pXO2^−^ *B*. *anthracis* Sterne. Our future work would utilize complementation of the wild-type Ant^−^ strains with functional anthrose operons as comparators to the homologous wild-type Ant^−^ strains in cell infection and vaccination studies using the Sterne and Biothrax vaccines in relevant animal models. Genetic and pathogenic characterization of anthrose-deficient anthrax indicates a potential threat to animal and human health. The effects of further adaptation and spread of emergent anthrose-deficient anthrax strains into new geographic areas is unknown but could amplify the global animal and human anthrax threat. Convergent evolution toward an anthrose-free exosporium could be ongoing in other geographically distant anthrax regions, and surveillance should be balanced appropriately.

## Materials and methods

### Bacterial strains, cell lines, and growth conditions

*Escherichia coli* DH5α was used as a cloning strain and grown in LB broth or agar at 37°C, and RHO3 was used as a mobilization strain and grown in the presence of 200 μg/ml of DAP (Sigma-Aldrich, USA; [78]). Kanamycin was used at 35 μg/ml in *E*. *coli* and 20 μg/ml in *B*. *anthracis* Sterne. Spectinomycin was used at 50 μg/ml in *E*. *coli* and 100 μg/ml in *B*. *aanthracis*. Conjugations were carried out as previously described [78,79]. *B*. *anthracis* Sterne 34F2 was obtained from Colorado Serum (USA) and grown with brain heart infusion (BHI) broth or agar at 37°C. Wild-type *B*. *anthracis* are from the Martin E. Hugh-Jones *Bacillus anthracis* Collection housed at the Emerging Pathogens Institute at the University of Florida. Bacteria were manipulated using BSL3 practices and procedures according to the Biosafety in Microbiological and Biomedical Laboratories (BMBL) in a CDC-/USDA-inspected and registered entity. A549 human lung epithelial cells and murine RAW264.7 macrophages were acquired from ATCC and maintained in DMEM+10% heat-inactivated FBS with antibiotic-antimycotic at 37°C and 5% CO_2_.

### Whole genome sequencing and bioinformatic analysis

gDNA was isolated using the QIAGEN DNeasy UltraClean Microbial Kit (Hilden, Germany), filter sterilized, and sterility verified according to internal procedures and CDC SAP guidance. gDNA was sheared, and libraries were produced with the NEBNext Ultra II Library Prep kit (NEB, Ipswich, Massachusetts, USA). The libraries were sequenced with the miSeq next-generation sequencing system (Illumina, San Diego, USA) at the UF EPI using v3 chemistry. Reads were quality controlled using standard methods, and SPAdes [80] was used for de novo genome assembly, while BWA-MEM [81] was used to align reads to *B*. *anthracis* Ames. Assembled genomes were analyzed using CLC Sequence Viewer 7.5.0 (QIAGEN), and alignments were viewed with Integrative Genomics Viewer version 2.5 (IGV; Broad Institute [82]). References sequences were obtained from GenBank. Whole genome SNP alignments were created with the PhAME software package developed at Los Alamos National Labs [83], and phylogenetic trees were generated with RAxML [84] within PhaME and visualized with iTOL [85]. BioSample accession numbers of the Illumina read files for UF00242 and UF00910 are SAMN15046868 and SAMN15046933 and have been deposited in BioProject PRJNA635616.

### PCR and molecular methods

PCR was carried out using standard methods [86] and Q5 *Pfu* polymerase (NEB). Splicing by overlap extension (SOE) PCR was carried out by first amplifying 1 kb of each of the regions flanking the *antC* gene. These fragments were combined in a 15-cycle PCR without primers. A total of 5 μl of the initial SOE reaction was used as template with end primers in a 30-cycle PCR. The resulting approximately 1.9-kbp fragment was digested with *Bam*HI and *Hind*III for further cloning as described below. PCR primers for genomic deletion verification were designed from the de novo assembled sequences of *B*. *anthracis* Sterne, UF0242, and UF0910.

### Simulated mutational acquisition studies

The simulations were performed using the University of Florida Research Computing Environment and the HiPerGator 2.0 supercomputer cluster [87]. Simulations were performed in annual discrete time slots over the period of 600 years. Annual time slots were selected due to low mutation activity in shorter time slots (days, weeks, or months) and the predominantly dormant lifestyle of *B*. *anthracis* in the spore state. For this work, we focused on a 2,000-bp region of *B*. *anthracis* gDNA containing a segment of the anthrose biosynthetic operon known to accumulate nonsense mutations that will inactivate one of the biosynthetic genes and prevent deposition of anthrose onto the surface of the spore. Within the computer simulation, this piece of DNA was identically replicated 10,000 times in computer simulation to create a synthetic population. This synthetic population was investigated for mutations, insertions, and deletions for the period of 600 years. We initially observed for the AAAAAAAGAAAAAAAG SSR triplication event (AAAAAAAGAAAAAAAGAAAAAAAG creating a stop) in *antC* of WAG *B*. *anthracis* [4,6]. The anthrax mutation probability per generation for SSR mutations is much higher than individual base pair mutation probabilities due to the mechanisms of polymerase slippage during genome replication and has been calculated previously (between 10^−4^ and 10^−5^ for SSR compared to 5.2 × 10^−10^ for point mutations) [88,89]. Therefore, the SSR mutation probability per generation was always assumed to be 10^4^ higher than the individual base pair mutation probability used in the simulation, and those mutations were considered first at every time step of the simulation process before the individual base pair mutations. The individual base pair mutation probability value, denoted as *λ*, for *B*. *anthracis* was derived from the literature [59,88], and individual base pair mutations were considered second after SSR mutation within every time step of the simulation process. This mutation rate per generation was adjusted by the multiplier *g* resulting in mutation probability *λ* × *g*. This multiplier *g* is determined by the number of generations per unit of time within the simulation (i.e., year). Within the simulation, the mutations in each time slot are assumed to be independent from other time slots. The logic behind this probability comes from the assumption of the Poisson process for the mutation counts with independent time slots. This results in the expected number of mutations equal to *λ* × *g* which serves as a binary probability of a single mutation in the given time slot. Within the simulation study, every seventh year was approximated to be epizootic [57], and during epizootic years, 43 generations of *B*. *anthracis* were assumed to occur [59] with just a single generation per year for sporadic years. The resulted multiplier values were *g* = 43 and *g* = 1, respectively.

In the simulation study, 10 different scenarios were considered, and the probability parameter *λ* × *g* was used in baseline simulation scenario 01 (sc01) for mutations, insertions, and deletions of individual base pairs. For each subsequent scenario, the parameter *λ* × *g* was increased by multipliers 100 (sc02), 200 (sc03), 300 (sc04), …, 800 (sc09) and by multiplier 1,000 for the last 10th scenario (sc10). To produce stable estimates 1,000 simulation replications were generated for each of 10 scenarios. The mutation probabilities were assumed to be the same for wild-type and mutant *B*. *anthracis*. The simulations for each scenario were performed independently for each element of the synthetic population. The uncertainty estimates were summarized across 1,000 replications for each scenario, and summary statistics were computed for each simulation replication within each simulation scenario, and uncertainty estimates were presented in S1 and S2 Tables in the form of quantiles across these 1,000 replications.

In S1 and S2 Tables, the column with 0% corresponds to the smallest statistic value across 1,000 replications for the given scenario, while 100% corresponds to the largest. The column with 50% corresponds to the median value of the statistic of interest, while 2.4% and 97.60% percentiles correspond to the 95% confidence interval for the given statistic. Input sequences, documentation, code, analysis, and data for these simulations are available at https://github.com/akirpich-ap/anthrax-simulations.

### Mutant generation and complementation

The anthrose-deficient Sterne strain was created using previously published methods [90]. Briefly, SOE PCR generated the Δ*antC* fragment for cloning into pRP1028 by *Bam*HI/*Hind*III digest. The temperature-sensitive plasmid was transformed into RHO3 then mated into Sterne overnight at 30°C on BHI+DAP. Colonies containing the allelic replacement plasmid were streaked on BHI-Sp100 and incubated at 37°C to select for chromosomal integration. Red fluorescence was verified by viewing under fluorescent excitation, and colonies were restreaked. Mutants were resolved by introducing I-*Sce*I-expressing plasmid pRP1099 into merodiploids and plating on BHI-Km20 as above using RHO3. Colonies no longer expressing RFP were PCR screened for mutation of *antC* then streaked on BHI to cure pRP1099. Deletion mutant colonies were resuspended in 95% ethanol and incubated for 1 hour to ensure that saved isolates retained the ability to sporulate after several rounds of culturing.

For complementation of the Δ*antC* mutant, the *antC* gene was amplified by PCR to add NheI and PstI sites, restriction digested with NheI and PstI and cloned into pRP1099 cut with the same enzymes to produce pRepU-Kan-*antC*, and plasmid confirmed by NheI/PstI digest. The complementation vector was transferred into *Ba* Δ*antC* by conjugation using RHO3 as previously described [78], followed by selection on kanamycin and PCR verification of plasmid presence.

### Spore generation and purification

Starter Sterne cultures were inoculated into 3 ml of BHI with shaking at 37°C overnight. The next day, 200-μl aliquots were plated on Difco sporulation media (DSM) agar. The cultures were incubated at 30°C for 5 days. The sporulation efficiency of each plate was checked by microscopy and was always ≥99 complete prior to harvesting in 2 ml of ice-cold sterile Milli-Q water. Spore preparations from multiple plates were combined, washed 2 times in ice-cold water, then purified through a diatrizoic acid gradient. The spore suspensions were resuspended in 95% ethanol to kill any residual vegetative cells, incubated for 1 hour with occasional vortexing, then washed 3 times in ice-cold sterile Milli-Q water. Aliquots were frozen at −80°C and periodically thawed, diluted, plated, and colonies enumerated to determine viable spores. Multiple spore preparations were used for the work in this manuscript. Spore were visualized under phase contrast microscopy using an EVOS XL Core Imaging System (Thermo Fisher Scientific, Waltham, Massachusetts, USA). Spores appear as phase bright points.

### Methanolysis, monosaccharide derivatization, and GC–MS of spore preparations

The 1 × 10^8^ spores of the indicated strains or pure anthrose (Sigma-Aldrich, St. Louis, Missouri, USA) were resuspended with 1 M methanolic HCl in glass bottles with TFPE lids and incubated for 16 hours at 80°C [91]. The samples were dried in a speed vac. This was repeated twice with 100% methanol. TMS derivatization was carried out using TMSI (Sigma-Aldrich; St. Louis, Missouri, USA) according to the manufacturer’s instructions. GC–MS analysis was carried out at the Mass Spectrometry and Education Center in the Department of Chemistry at the University of Florida.

### Growth curve analysis

Growth curves were carried out by growing starter cultures overnight in BHI at 37°C with shaking. The cultures were diluted to an OD_600_ of 1 then inoculated into modified G media or BHI at a 1:100 dilution. Growth curves were carried out in a Biotek Synergy H1 96-well plate reader in triplicate with orbital shaking at 425 cpm, and the OD_600_ was read every 5 minutes.

### Germination and sporulation assays

Germination assays were carried out by thawing frozen aliquots of spores, prepared as above, on ice then diluting to 1 × 10^9^ spores/ml in ice cold 1X PBS. The spore suspensions were heat-activated for 30 minutes at 70°C, then inosine or L-alanine was added to a final concentration of 1 mM or 60 mM, respectively. The OD_600_ was measured every 2 minutes at 37°C preceded by shaking for 5 seconds. The assay was carried out in biological triplicate and technical duplicate. Germination of each strain was normalized to the starting OD_600_ of each strain and is expressed as percentage of starting OD_600_.

For sporulation assays, strains were grown to stationary phase in BHI at 37°C and shaken at 225 rpm. OD_600_ was adjusted to 1 in BHI and used at 1:100 to inoculate flasks of modified G medium in triplicate for each strain [92] followed by incubation at 30°C and shaking at 225 rpm. For ethanol treatment spore counting at 24 and 72 hours, 50-μl aliquots were resuspended in 1 mL of ice cold 90% ethanol and kept on ice for 1 hour, being vortexed every 15 minutes for 15 seconds. Samples were centrifuged for 30 minutes at 4°C at 25,000 × g. Pellets were suspended in PBS + 0.05% Tween-20 (PBS/T), serially diluted, and plated on Bacto BHI agar. For colony counting, untreated aliquots were suspended in PBS/T, serially diluted, and plated on Bacto BHI agar. Plates were incubated at 37°C overnight, after which colonies were counted. The data shown are the average of 2 independent experiments carried out in triplicate. For heat-inactivation spore enumeration, cultures were grown as above and two 1-ml aliquots were removed every 24 hours for 3 days and dilution plated on BHI or heated at 95°C for 10 minutes then dilution plated, as described previously [93], to determine the number of spores and total bacteria present in the cultures. The experiment was carried out in triplicate.

### Cellular spore association assay

A549 lung epithelial cells or RAW264.7 macrophages were seeded at 1.5 × 10^5^ cells/well in 24-well Corning CellBIND plates and allowed to attach overnight. The next day, monolayers were washed 3 times with 1X PBS. Spores were thawed on ice and quickly diluted to a multiplicity of infection (MOI) of 10:1 in 200 μl DMEM+10% heat-inactivated FBS, added to the monolayers, and incubated at 37°C in 5% CO_2_ for 1 hour. After 1 hour, the supernatants were removed, monolayers were washed 3 times with 1X PBS, then media with 10 μg/ml of gentamicin was added for 1 hour to kill extracellular bacteria. Monolayers were washed 3 more times with 1X PBS to remove antibiotics and any uninternalized spores, then cold water was added to lyse cells, and the suspensions were incubated for 15 minutes then dilution plated on BHI to count the number of internalized bacteria. The initial inoculums were dilution plated to count the number of spores used to infect the monolayers and to calculate internalization efficiency per strain which is presented in relation to Sterne. This experiment was carried out in triplicate in 2 independent experiments. The data were averaged from the 6 data points, and the standard deviation was calculated.

### *Galleria mellonella* infection study

*G*. *mellonella* were purchased from Carolina Biological Supply (Burlington, North Carolina, USA). In total, 100 to 250 mg worms were used in groups of approximately 25 Sterne, and Δ*antC* mutant spores were generated and purified as described above. Purified spores were stored at −80°C, thawed, and enumerated by dilution plating before diluting down to the desired inoculum of 10^7^ spores/ml in PBS. Spore dilutions were kept on ice, and a 27-gauge needle attached to a Hamilton 50-μl microsyringe was used for injections. Larvae were briefly placed on ice then held with forceps to inject 10 μl (10^5^ spores) into the fifth larval abdominal section containing the third distal set of prolegs. Worms were observed at room temperature for 30 minutes before being placed at 37°C. Worms were checked for survival every 8 hours over the 72-hour study by gentle prodding for movement.

### Animal studies

Four to 6-week-old female A/J mice were purchased from The Jackson Laboratory (Bar Harbor, Maine, USA). Animals were housed in microisolator cages under pathogen-free conditions with food and water ad libitum. Spores were produced, purified as above, and frozen in 20% (v/v) glycerol aliquots at −80°C. An aliquot of each was thawed and spores enumerated by dilution plating on BHI. Dilution values were determined, and aliquots were thawed on ice and diluted with cold 1X PBS for target inoculation in 100 μl of PBS for subcutaneous injections or 20 μl of PBS for intranasal instillation. Groups of 5 mice (*n* = 5) were challenged by subcutaneous injection with 100-μl inoculum containing 10^0^, 10^1^, 10^3^, 10^4^, or 10^6^ spores. The same was done for intranasal instillation of 20 μl, but mice were first anesthetized with intraperitoneal ketamine/xylazine injection. In 2 independent studiess, 10 mice in 2 additional experiments were infected subcutaneously with 10^3^ spores of each strain and were used to confirm our observations of decreased TTD and lower survival percentages near the LD_50_ dose. The numbers from all experimental challenges were combined for total of 15 mice in Fig 4A. For the predetermined endpoint study, groups of 5 mice were humanely euthanized at 48 hours following 10^3^ spores subcutaneous challenge. Spleens were removed and processed in 5 ml of 1X PBS using a stomacher (Interscience, Saint-Nom-la-Breteche, France, EU). Undiluted and diluted aliquots were plated on BHI for CFU determination. Mice were observed thrice daily for the first 4 days then once daily until the end of the 14- or 21-day studies. Mice were euthanized when moribund or at the end of the study. LD_50_ values were calculated by Probit regression using the Stata software package (StataCorp, College Station, Texas, USA).

Prime boost vaccination studies were carried out similarly as the LD_50_ studies, but mice were primed by intramuscular injection of 50 μl (25 μl in each caudal thigh muscle) with the FDA-approved BioThrax anthrax vaccine adsorbed (AVA) vaccine (BEI Resources) diluted 1:4 in PBS and then boosted 2 weeks later. Four weeks post-boost, groups of 5 mice were subcutaneously challenged with 20, 50, or 100 times the LD_50_ of each strain calculated for *B*. *anthracis* Sterne in this study, and survival was observed out to 21 days. These challenge doses were associated with vaccine failure in mice when using wild-type *B*. *anthracis*.

### Competitive index study

To ensure pRepU-kan-*antC* was maintained by complemented spores, 1 × 10^7^ CFU Δ*antC*/COMP spores were inoculated in 3 ml of BHI in triplicate in the absence of kanamycin and grown with shaking at 37°C for 24 hours. After 24 hours, the cultures were dilution plated on BHI and BHI+Km20. CFU counts indicated no significant difference between kanamycin-resistant colonies on BHI+Km20 and total colonies on BHI. This means that the plasmid was stable after 24-hour growth at 37°C in the complement strain, facilitating the in vivo CI study. Here, the in vitro CI was determined by inoculating the Δ*antC* and Δ*antC*/COMP mixed in a total of 2 × 10^7^ spores and inoculated into 3 ml of BHI in triplicate. The cultures were incubated at 37°C with shaking for 24 hours, then diluted in PBS/T and plated for CFU counts on BHI and BHI+Km20. The calculated CFU/ml on BHI+Km20 (the Δ*antC*/COMP concentration) were subtracted from the calculated CFU/ml on BHI to obtain the Δ*antC* concentration. The in vitro CI was calculated by the following equation: (CFU/mlofBaΔantC)÷(CFU/mlofBaΔantC/COMP). For the *in vivo* CI, ten 4- to 6-week-old female A/J mice were injected subcutaneously with 2 × 10^7^ spores (a mixture of Δ*antC* and Δ*antC*/COMP) in 100 μl of 1XPBS. Twenty-four hours later, mice were humanely killed, and the kidneys, liver, and spleen of each mouse were pooled in 5 ml of PBS/T. The organs were homogenized with a handheld homogenizer, diluted in PBS/T, with each dilution plated on BHI and BHI+Km20. The *in vivo* CI was calculated as described above for the *in vitro* CI.

### Pure death process simulation studies

We used a log-rank test to test for significant differences in TTD between wild-type and anthrose-negative populations. These tests detect differences in patterns regardless of the nature of the process. To test biological hypotheses on virulence and pathogenicity of our mutant and wild-type strains, we modeled the subcutaneous infection assays with a pure death process [53], whose mechanistic approach is ideal for testing such hypotheses. Fitting this model via maximum likelihood is the best approach in our case because the exact time of death of every mouse is never recorded. Additionally, the model considers any possible dependencies in the infection process for mice sharing a cage.

#### Stochastic models experiment: Death in mice and larvae

To model the experimental infection death process in both mice and *Galleria* larvae, we used a “stochastic death process” [94], which is a well-known continuous time and discrete states Markov process [95] used in various epidemiological contexts [95–98], notably to model Hanta virus deaths [99]. It is also the same type of stochastic process used to estimate the time til the most recent common ancestor of highly infective viral variants [100] and the time from infection to death by *B*. *anthracis* in zebra [101].

Let *N*(*t*) be the number of mice alive at time *t* for a single experimental batch. We denote the initial number of infected mice in an experimental assay, *N*(0), as *m*. In our experiments, usually, *m* = 5. Heuristically, the death process can be described as a process where mice are being lost one at a time, at random time points since the beginning of each trial. Deaths occur at a rate *μ*_*n*_ and at random time intervals. One of the key characteristics of this stochastic process that makes it suitable to model the experimental deaths is that it is not necessary to record the exact moment at which every single death occurs, and observations can be made at unequal sampling intervals. The only data needed to fit different models for the death rate are the number of mice alive at different time points. We note in passing that here we use the convention that random variables are written with capital letters and realized values as lowercase letters. Hence, *n*(*t*) denotes the observed and remaining number of mice at time *t* and thus, 1 realization of the random variable *N*(*t*).

The death process can be formulated so that it embodies a suite of plausible biological hypotheses regarding the unfolding of the infection process, and in particular, the dependency of its intensity on biological factors of interest, like the nature of the inoculant (i.e., wild type or mutant). Not only can the mice loss rate be specified as a function of the total amount of mice present in the batch, but also it can be written in standard regression format as a function of 1 or more discrete or categorical variables. The categorical variable of interest here was the strain (wild type, mutant, or complement), and the continuous variable of interest was the infection dose (in CFU units). Using standard stochastic process results, one may arrive at an analytical expression for the probability that at any given time *t*, the number of alive mice is equal to any number *n*(*t*), as a function of the death rate, which is, in turn, modulated by the categorical and quantitative variables of interest. We denote the probability of observing *n*(*t*) alive mice at time *t* as Pr(N(t) = n(t)) as *p*_*n*_(*t*). The resulting expression for *p*_*n*_(*t*) under each biological hypothesis served as the direct link that connected the observations with the proposed probabilistic model.

The general death process formulation applied to our case assumes that there exists an arbitrarily small amount of time *Δt* during which at most 1 mice dies from the *B*. *anthracis* infection with probability *μ*_*n*_(*Δt*) and that the probability that no loss by death occurs is 1−*μ*_*n*_(*Δt*). Finally, it also assumes that the probability of any other event is negligible, i.e., that only deaths and not births can be observed. Accordingly, the probability that at time *t*+Δ*t* the total number of mice is equal to *n* is obtained as the sum of the probability that at time *t*, there were *n*+1 mice and a death occurred and the probability that at time *t*, there were already *n* mice and no loss occurred within Δ*t* units of time, i.e.,

$$p_{n}(t+\Delta t)=(\Delta t)\mu_{n+1}p_{n+1}(t)+(1-\mu_{n}(\Delta t))p_{n}(t).$$ (1)

As Δ*t*→0 and after a simple manipulation, this equation tends to the following system of ODEs:

$$\frac{dp_{n-1}(t)}{dt}=\mu_{n}p_{n}(t)-\mu_{n-1}p_{n-1}(t),$$

where *n* = *m*, *m*−1, *m*−2,…,2,1,0. Letting μ_*n*_ take on specific forms allows one to solve this system of ODEs to obtain expressions for *p*_*n*_(*t*). With these probabilities in hand, one describe the entire behavior of the process. For instance, one can find its time-dependent mean and variance. In particular, when μ_*n*_ is a linear function of *n*, the time-dependent mean of the process matches the solution of a deterministic ODE model counterpart. If for instance we let μ_*n*_ = μ*n*, then the mean of the stochastic model matches the solution of its deterministic counterpart, the ODE model $\frac{dn(t)}{dt}=-\mu n(t)$ for the number of alive mice at time *t*. To see why, first note that the initial conditions

$$p_{m}(0)=1,p_{n}(t)=0\;\mathrm{if}\;n>m\;\mathrm{or}\;n<0$$

allow us to solve the above system of ODEs to yield [51]

pn(t)=(mn)(e−μt)n(1−e−μt)m−n (2)

In other words, the number of mice at time *t*, *N*(*t*) is binomially distributed with probability of success e^−μ*t*^. This quantity denotes the probability that no death occurs during time the time period (0,*t*). According to the properties of the Binomial distribution, the time-dependent average algae size is given by E[*N*(*t*)] = *m*e^−μ*t*^ and its variance by V[*N*(*t*)] = *m*e^−μ*t*^(1−e^−μ*t*^). Hence, on average, the process behaves like its deterministic counterpart, an exponentially decaying total number of surviving mice. Its variability, however, is modulated by both, the death rate and the amount of elapsed time. To solve the system of ODEs above, one arrives first at an expression for the (random) total amount of deaths since the beginning of the process. This amount of deaths turns out to be binomially distributed with parameters *m* and 1−e^−μ*t*^ from which Eq 2 follows [96].

#### Fitting the death process model to data

The basic data unit consists of the observed pairs number of surviving mice or larvae and time:

$$(n_{0},t_{0}),(n_{1},t_{1}),(n_{2},t_{2}),\ldots ,(n_{q},t_{q}),$$

where *q*+1 is the total number of observed time points. Regarding each observation as the initial condition for the next one, from Eq 2 it follows that

Pr(N(ti)=ni|N(ti−1)=ni−1)=f(ni,ti−ti−1|ni−1)=(ni−1ni)(e−μ(ti−ti−1))ni(1−e−μ(ti−ti−1))ni−1−ni

Now, using the Markov property and letting τ_*i*_ = *t*_*i*_−*t*_*i*−1_ to simplify notation, the joint probability of the observations or likelihood function for the observations

$$(n_{0},t_{0}),(n_{1},t_{1}),(n_{2},t_{2}),\ldots ,(n_{q},t_{q}),$$

for 1 experimental batch can be written as

L(μ)=f(n1,τ1|n0)f(n2,τ2|n1)…f(nq,τq|nq−1)=∏i=1q(ni−1ni)(e−μτi)ni(e−μτi)ni−1−ni.

This formulation, however, assumes that the per-mouse death rate *μ* is a constant independent of dose. Hence, to incorporate our different biological hypotheses regarding the factors influencing the death rate, we wrote *μ* as a log-linear model of the categorical and continuous variables of interest. Specifically, to test the hypothesis that the death rate differed between the mutant (M) strain and the wild type (W) strain, we fitted a likelihood function with a different set of parameters governing the death rate of each strain, i.e., where the death rate of the mutants was

$$\log(\mu_{M})=\beta_{0}^{M}+\beta_{1}^{M}log(\mathrm{dose})$$

and that of the wild type was

$$\log(\mu_{W})=\beta_{0}^{W}+\beta_{1}^{W}log(\mathrm{dose})$$

and compared that fitting via the BIC with the model that stated that only 1 set of parameters was necessary to explain, both, the mutant strain trials and the wild-type trials with the *Joint* log-linear model

$$\log(\mu_{J})=\beta_{0}^{J}+\beta_{1}^{J}log(\mathrm{dose}).$$

The model with the lowest BIC was deemed as the best model [102]. From heretofore, we will denote the hypothesis that states that each strain imposes a different death rate as the “Separate dynamics model” and its null counterpart as the “Joint dynamics model”. Finally, we compared the fit of these 2 models by pooling all data across all doses as well as dose per dose. The results of every fit, the BIC scores for every model, and the conclusions of every test are shown in Table 2. The code, instructions, and data output for these analyses are freely available in https://github.com/jmponciano/mice.

**Table 2:** Results for the model selection test.

	Challenge modality	BIC null (Joint dynamics)	BIC alternative (Separate dynamics)			Strength of Evidence
Dose		M and W yield equal death rates	M has a faster death rate than W	Delta BIC	Best model	
All doses	Intranasal	51.77302	55.53519	3.76217	Equal death rates	Strong evidence
All doses	Subcutaneous	115.9206	121.1775	5.2569	Equal death rates	Strong evidence
100,0000 CFUs	Intranasal	29.53845	31.69928	2.16083	Equal death rates	Weak evidence
100,0000 CFUs	Subcutaneous	20.1946	22.86277	2.66817	Equal death rates	Weak evidence
10,000 CFUs	Intranasal	16.7423	20.32083	3.57853	Equal death rates	Strong evidence
10,000 CFUs	Subcutaneous	30.27522	34.16703	3.89181	Equal death rates	Strong evidence
1,000 CFUs	Intranasal	8.269192	8.876708	0.607516	Equal death rates	Weak evidence
1,000 CFUs	Subcutaneous	67.1648	70.25619	3.09139	Equal death rates	Strong evidence
10 CFUs	Intranasal	1.386294	2.772589	1.386295	Equal death rates	Weak evidence
10 CFUs	Subcutaneous	1.386294	2.772589	1.386295	Equal death rates	Weak evidence

BIC, Bayesian Information Criterion; CFU, colony-forming unit.

Determination of whether the mutant strains resulted in the same death rate than the wild-type strain (Null model or “Joint dynamics model”). If the difference in information criteria is greater than 3 points, the evidence supporting the conclusion (equal or different death rates) is strong and roughly corresponds to a significant hypothesis test at the 5% alpha level.

As shown in Table 2, none of the comparisons pointed toward a significant difference between the death rate of the mutant strain vis-à-vis the death rate of the wild-type strain. Using the data for the 1,000 CFUs dose, we plotted the average estimated loss rates for each strain under the “Separate dynamics model” (Fig 4C). This plot shows that although the mutants death rate is on average predicted to be faster than the death of the wild type, the confidence intervals of both average death trends overlap substantially.

This overlap of confidence intervals led us to investigate via simulations whether infection assays started with larger sample sizes and assumed to evolve according to the same estimated dynamics difference than the 1,000 CFU assays plotted in Fig 4C would yield significant differences. To do that, we simulated experimental assays in batches of 3 replicates, each started with initial sample sizes of 5, 10, 20, 40, and 80 mice using the Gillespie algorithm for stochastic processes[95], parameterized with the maximum likelihood estimates of the “separate model dynamics” for the 1,000 CFU data shown in Table 3.

**Table 3:** MLEs of the “separate model dynamics” for the 1,000 CFU mouse data in mice and larvae.

**Mice**				
**Strain**	**Parameter**	**2.50%**	**MLE**	**97.50%**
**Wild type**	Beta0	−0.0808	−0.0643	−0.0479
**Wild type**	Beta1	−0.5657	−0.4521	−0.3385
**Mutant**	Beta0	−0.0631	−0.0496	−0.0362
**Mutant**	Beta1	−0.442	−0.3492	−0.2563
**Larvae**				
**Strain**	**Parameter**	**2.50%**	**MLE**	**97.50%**
**Wild type**	Beta0	0.00928471	0.011138	0.01299171
**Wild type**	Beta1	−0.3257925	−3044529	−0.2831132
**Mutant**	Beta0	−0.0042344	−0.002506	−0.0007779
**Mutant**	Beta1	−0.2509442	−0.231046	−0.2111493

MLE, maximum likelihood estimate.

In summary, to determine the size of the population of mice or larvae to detect a difference of size equal to the one observed for subcutaneous challenge, we:

1. Simulated 1,000 data sets of the same dimension as the ones observed (3 batches of 5 mice followed for 15 days for each of the strains: wild type and Mutant) using a stochastic death process parameterized with the original data;
2. Estimated the death rate as a function of the dose for every simulated data set and for every strain;
3. Tested whether the estimated difference was significantly large using the BIC;
4. Computed the proportion of times the “true” difference was actually detected; and
5. Repeated steps 1 to 5 for data sets with 3 batches of 10, 20, 40, and 80 mice, respectively.

Results from the simulations were used to estimate the power of the infection assays in mice for each population size.

### Ethics statement

Animal studies were carried out at the USDA accredited University of Florida animal care facility under approved UF IACUC protocol number 201810488. Animals were housed in microisolator cages under pathogen-free conditions with food and water ad libitum. Mice were euthanized by CO2 asphyxiation followed by cervical dislocation using AVMA-approved methods at humane endpoints or at the end of the study. Ketamine and xylazine cocktail were injected intraperitoneally prior to intranasal spore instillation.

## Supporting information

S1 Fig Genetic and GC–MS confirmation of *B*. *anthracis* Sterne Δ*antC* mutant creation. (A) Agarose gel showing the shift in size after deletion of *antC* in the *Ba* Sterne 34F2 strain. (B) The GC retention times of TMSI derivatized pure anthrose, *Ba* Sterne spores, and *Ba* Sterne Δ*antC* spores. Peaks of interest are indicated by red box. (C) Mass spectrum of the peaks indicated in (B) show the unique spectrum of pure anthrose matches the spectrum found in *Ba* Sterne spores and is not found in the *Ba* Sterne Δ*antC* spore spectrum. (D) The structure of TMSI derivatized anthrose deduced from the spectra in (C). The data underlying S1B and S1D Fig can be found in S1 Data. GC–MS, gas chromatography–mass spectrometry.
(TIF)

S2 Fig Survival curves of groups of 5 A/J mice challenged with *Ba* Sterne, *Ba* Sterne Δ*antC*, and *Ba* Sterne Δ*antC/*COMP spores. Ketamine-/xylazine-anesthetized mice were challenged by intranasal instillation and monitored for survival (left column). The 10^6^ spore intranasal challenge is presented in the main article. Mice were challenged by subcutaneous injection with the indicated doses (right column). The 10^3^ spore subcutaneous challenge is presented in the main article. The data underlying S2A–S2H Fig can be found in S1 Data.
(TIF)

S3 Fig Microscopy of spores and protective efficacy of AVA in the A/J mouse model. (A) Two representative phase contrast images of purified spores as prepared for mouse challenges and cellular studies. Magnification is 200×. (B) Survival of unvaccinated (blue lines) challenged with 20 times the LD_50_ and vaccinated (red lines) mice challenged with 20, 50, or 100 times the LD_50_ of *Ba* Sterne, *Ba* Sterne Δ*antC*, or *Ba* Sterne Δ*antC* COMP. The data underlying S3B Fig can be found in S1 Data. AVA, anthrax vaccine adsorbed.
(TIF)

S1 Raw Images Unedited agarose gel of data presented in Fig 2D and 2E and S1A Fig. The original gel image was cropped and separated for inclusion in Fig 2D and 2E. This original gel image was cropped and inverted for inclusion in S1A Fig.
(PDF)

S1 Table Summary quantiles of the estimated bacteria population proportion. Summary quantiles of the estimated bacteria population proportion of duplex regions in *antC* that became triplex stop mutants over the simulation period for ten different simulation scenarios. The table column with 50% corresponds to the estimated values, while 2.4% and 97.6% columns correspond to the confidence interval bounds.
(XLSX)

S2 Table Summary quantiles of the estimated average population base pair differences. Hamming distance from the beginning of the simulation period for 10 different simulation scenarios. The table column with 50% corresponds to the estimated values, while 2.4% and 97.6% columns correspond to the confidence interval bounds. Simulation was inclusive of the 8-bp *antC* SSR mutation and point mutations over the 600-year simulation.
(XLSX)

S1 Data Raw data for figures throughout the manuscript. Fig 1C_SNPalignments: Data presented here are the genome wide SNP alignment sequences for each strain as determined by the PhAME alignment software. Fig 1D_RAxML bootstrapcalcs: from the RAxML bootstrap analysis. The linkages generated from each of the 100 boot strapping repetitions are listed and are the source of the boot strapping values presented in Fig 1D. The bipartitioned tree produced from these is presented in Fig 1D. Fig 1F: We have provided the means and upper/lower limits generated by the simulation code. The input sequence and code are available at the github link indicated in the manuscript to enable reproduction of our simulation. Raw data output from 1,000 simulation runs each containing 10,000 input DNA sequences across 10 different scenarios generated a huge amount of raw data output which the code processes into the observed means and error. Fig 1G: The mean Hamming distance as a function of population percentage is presented. Using the code and sequence input from https://github.com/akirpich-ap/anthrax-simulations, the mean Hamming distance and proportion of bacterial sequences acquiring a stop codon can be reproduced. The Hamming distance represents the nucleotide sequence deviation from the input. Fig 3A: OD600 measured at T = 0 and every 2 minutes and used to calculate % of starting OD600. The strains and replicates are listed. Fig 3B: Raw CFUs, spore counts, and statistical methods for calculating significance are presented. Data for all subpanels are shown. Fig 3C: Spore association with each cell type as a % of wild type are indicated. Individual data points from 2 experiments are presented with the mean and SD in the Fig 3C. Fig 3D: raw mouse survival data following intranasal challenge with 1 × 10^6^ spores. One indicates a mouse death at a certain day, and a 0 indicates survival until study endpoint. Fig 3E: CFU counts for each mouse challenged singly with spores of the different strains. Each individual data point, the means and SD are shown in the original Fig 3E. Fig 3F: CFU counts of mutant and mutant complement bacteria in pooled organs of the same mouse are listed. Fig 3G: CFU counts for in vitro and in vivo studies and calculations of the CI for each replicate are presented. Fig 4A: raw mouse survival data following subcutaneous challenge with 1,000 spores. One indicates a mouse death at a certain day, and a 0 indicates survival until study endpoint. Fig 4B: raw *Galleria mellonella* survival data following spore challenge. One indicates a mouse death at a certain day, and a 0 indicates survival until study endpoint. Fig 4C–4G: Simulation code, input, and output files are available at https://github.com/jmponciano/mice. S1B and S1D Fig: The images in S1B and S1D Fig are the raw readouts from the gas chromatography mass spectrometer and were not modified in any way. From these raw readouts, data about peak area and observed mass spectra were extracted and are presented here. S2 Fig: raw mouse survival data following intranasal (left column) and subcutaneous (right column) spore challenge. One indicates a mouse death at a certain day, and a 0 indicates survival until study endpoint. S3B Fig: raw mouse survival data following vaccination with AVA and subcutaneous spore challenge of the indicated strains at the indicated doses. One indicates a mouse death at a certain day, and a 0 indicates survival until study endpoint. AVA, anthrax vaccine adsorbed.
(XLSX)

## Abbreviations

AVA: anthrax vaccine adsorbed
BIC: Bayesian Information Criterion
BHI: brain heart infusion
BMBL: Biosafety in Microbiological and Biomedical Laboratories
CFU: colony-forming unit
CI: competitive index
EF: edema factor
DSM: Difco sporulation media
FDA: Food and Drug Administration
GC–MS: gas chromatography–mass spectrometry
IgG: immunoglobulin G
IgM: immunoglobulin M
LF: lethal factor
MLVA: multi-locus variable number tandem repeat
MOI: multiplicity of infection
ODE: ordinary differential equation
PA: protective antigen
qPCR: quantitative PCR
sc01: scenario 01
SOE: splicing by overlap extension
SSR: simple sequence repeat
TMS: trimethylsilyl
TTD: time to death
WAG: West African Group
