# Correction: Convergent evolution of diverse *Bacillus anthracis* outbreak strains toward altered surface oligosaccharides that modulate anthrax pathogenesis

**DOI:** 10.1371/journal.pbio.3001112

**Published:** 2021-02-08

**Authors:** Michael H. Norris, Alexander Kirpich, Andrew P. Bluhm, Diansy Zincke, Ted Hadfield, Jose Miguel Ponciano, Jason K. Blackburn

The second author’s affiliation was listed incorrectly. The correct affiliation for Alexander Kirpich is:

**3** Department of Population Health Sciences, Georgia State University, Atlanta, Georgia, United States of America
