# Supplementary figures and images for: Convergent evolution of diverse Bacillus anthracis outbreak strains toward altered surface oligosaccharides that modulate anthrax pathogenesis
Source: PLoS Biol. 2020 Dec 28;18(12):e3001052. doi: 10.1371/journal.pbio.3001052 (PMC7793302; doi:10.1371/journal.pbio.3001052)

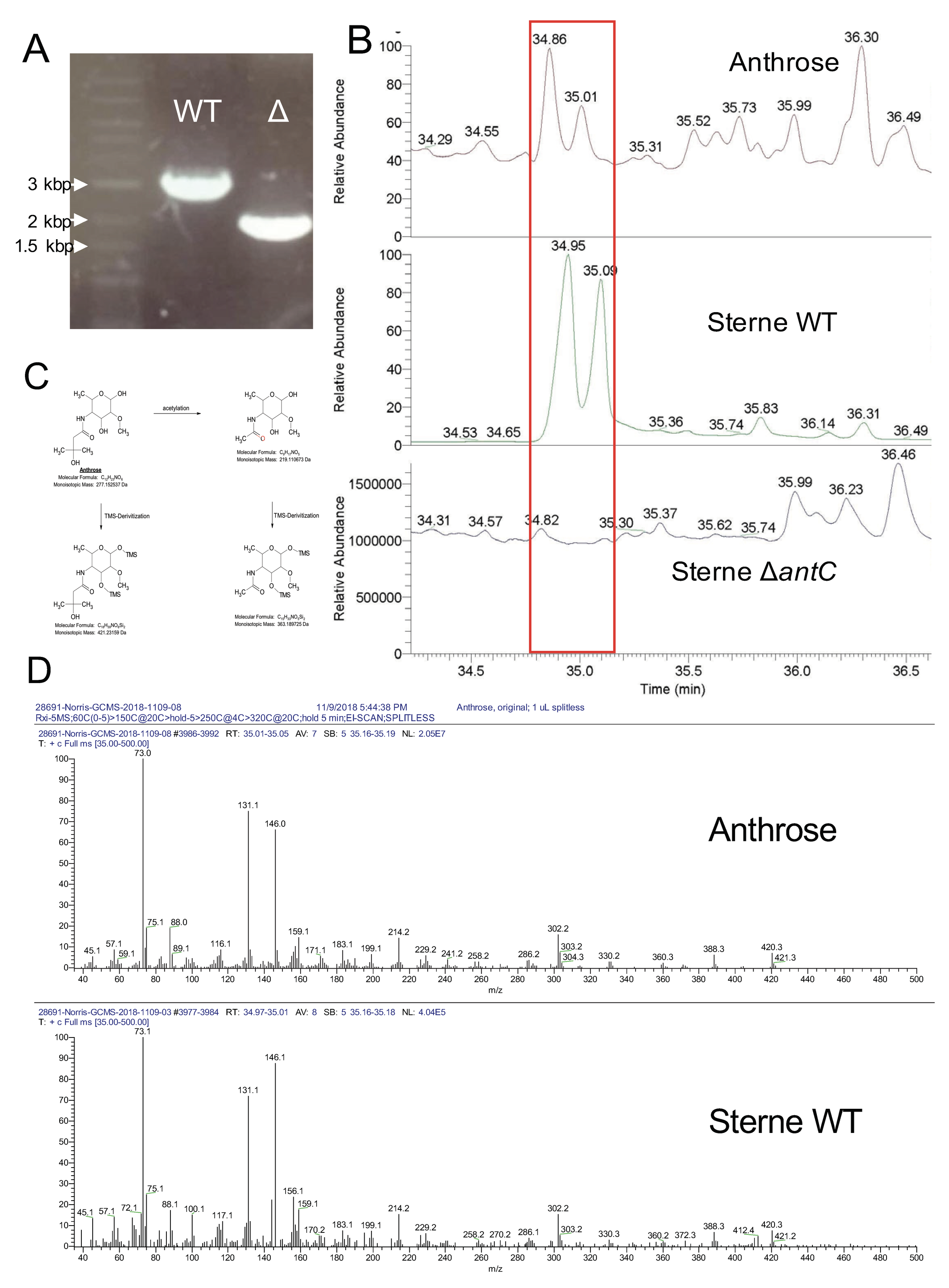

Supplement: S1 Fig — (A) Agarose gel showing the shift in size after deletion of antC in the Ba Sterne 34F2 strain. (B) The GC retention times of TMSI derivatized pure anthrose, Ba Sterne spores, and Ba Sterne ΔantC spores. Peaks of interest are indicated by red box. (C) Mass spectrum of the peaks indicated in (B) show the unique spectrum of pure anthrose matches the spectrum found in Ba Sterne spores and is not found in the Ba Sterne ΔantC spore spectrum. (D) The structure of TMSI derivatized anthrose deduced from the spectra in (C). The data underlying S1B and S1D Fig can be found in S1 Data. GC–MS, gas chromatography–mass spectrometry. (TIF) [file pbio.3001052.s001.tif]

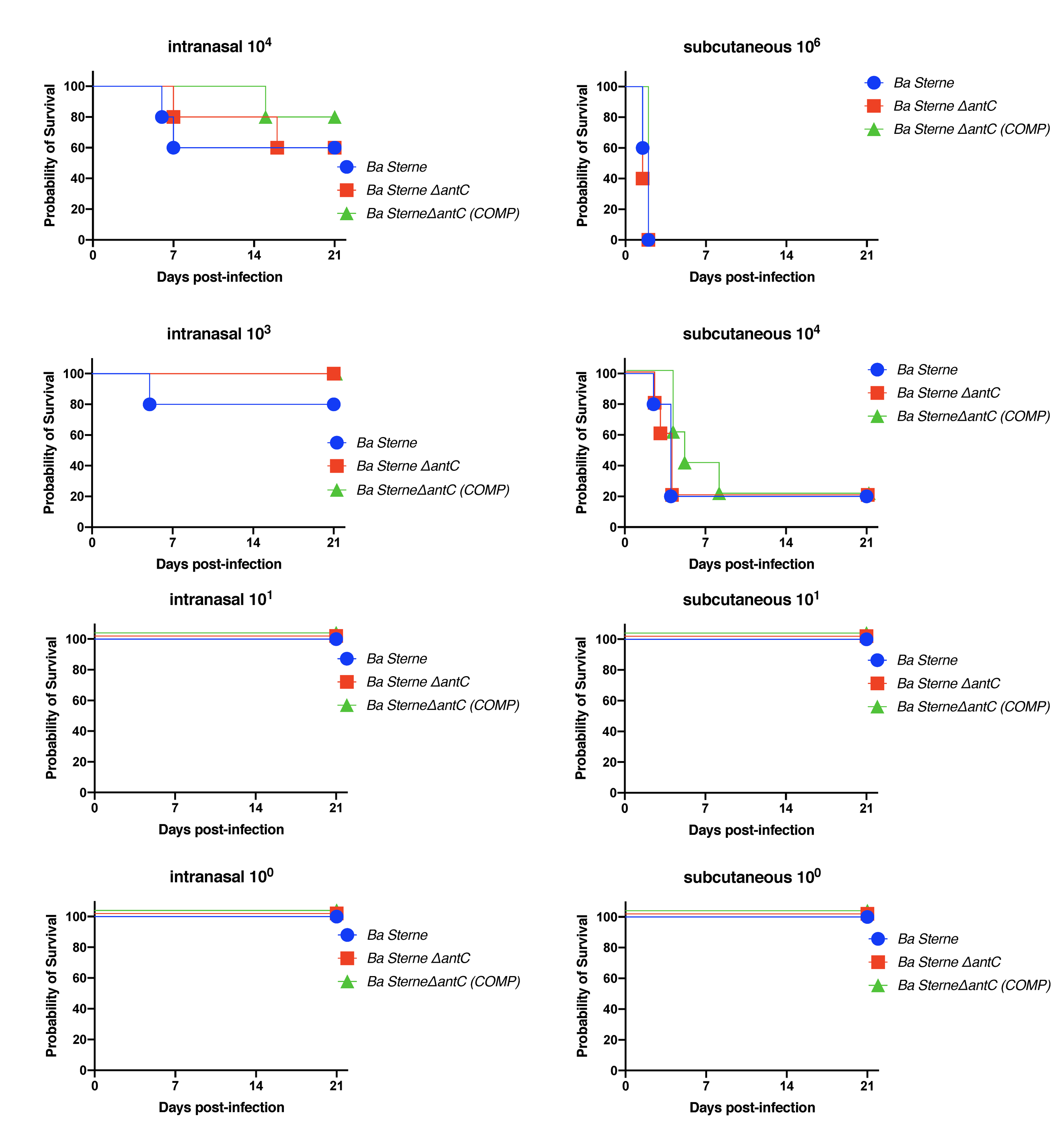

Supplement: S2 Fig — Ketamine-/xylazine-anesthetized mice were challenged by intranasal instillation and monitored for survival (left column). The 106 spore intranasal challenge is presented in the main article. Mice were challenged by subcutaneous injection with the indicated doses (right column). The 103 spore subcutaneous challenge is presented in the main article. The data underlying S2A–S2H Fig can be found in S1 Data. (TIF) [file pbio.3001052.s002.tif]

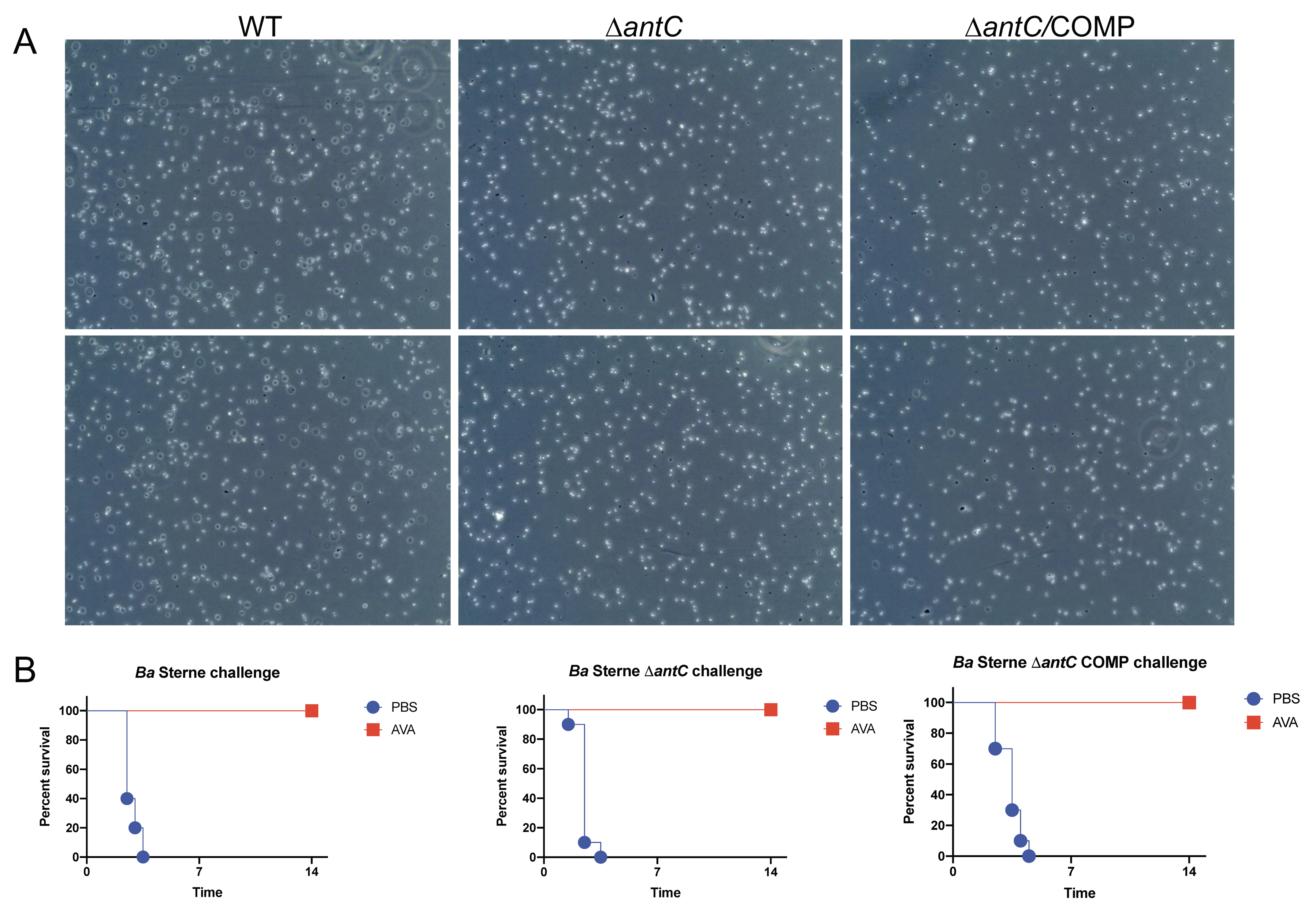

Supplement: S3 Fig — (A) Two representative phase contrast images of purified spores as prepared for mouse challenges and cellular studies. Magnification is 200×. (B) Survival of unvaccinated (blue lines) challenged with 20 times the LD50 and vaccinated (red lines) mice challenged with 20, 50, or 100 times the LD50 of Ba Sterne, Ba Sterne ΔantC, or Ba Sterne ΔantC COMP. The data underlying S3B Fig can be found in S1 Data. AVA, anthrax vaccine adsorbed. (TIF) [file pbio.3001052.s003.tif]

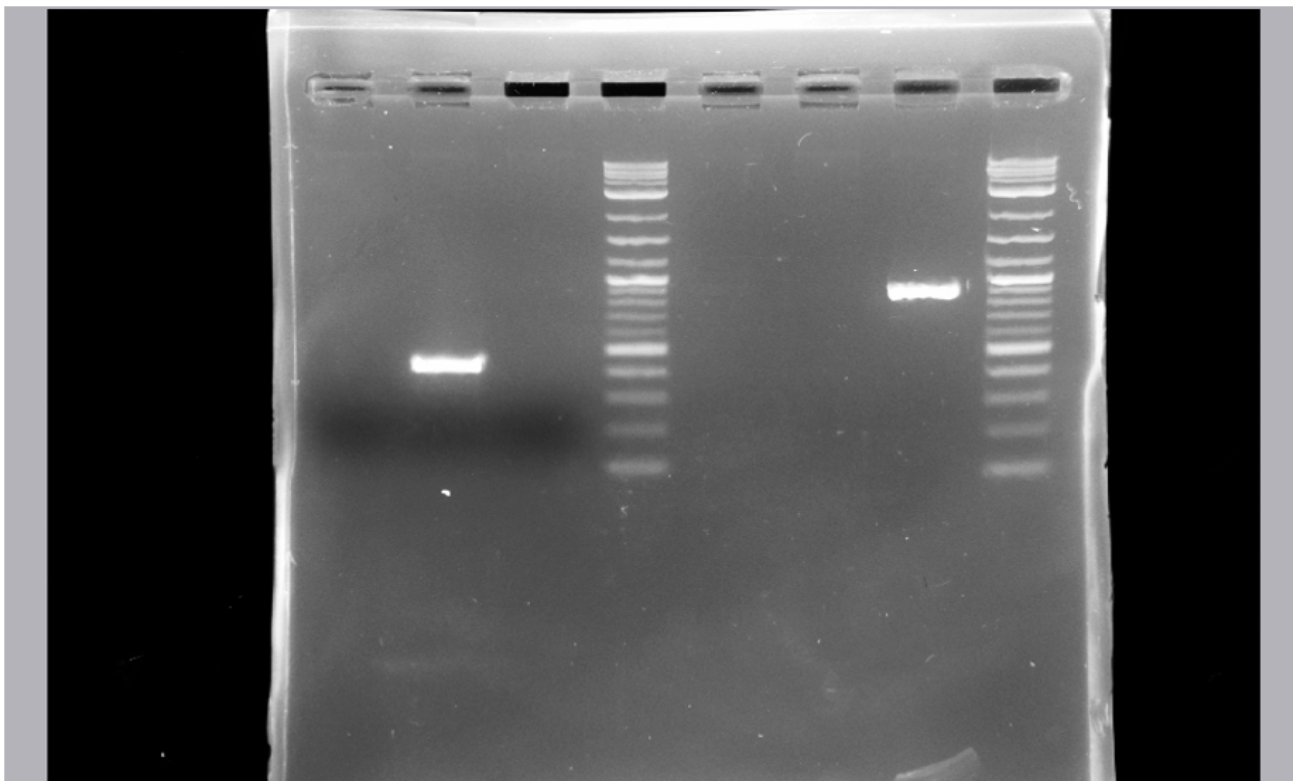

Fig 2D and 2E

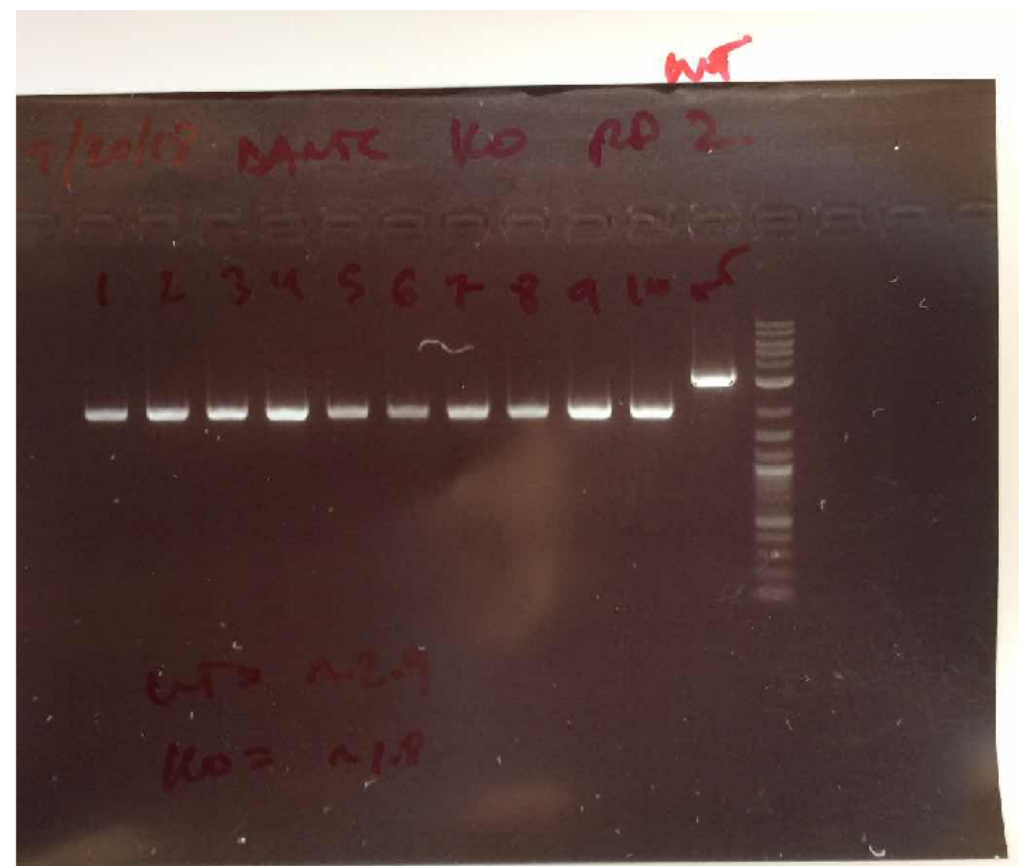

S1 Fig.

Supplement: S1 Raw Images — The original gel image was cropped and separated for inclusion in Fig 2D and 2E. This original gel image was cropped and inverted for inclusion in S1A Fig. (PDF) [file pbio.3001052.s004.pdf]
